# Dynamics of Deleterious Mutations and Purifying Selection in Small Population Isolates

**DOI:** 10.1093/molbev/msaf110

**Published:** 2025-07-21

**Authors:** Ying Chen, Xueyun Feng, Kerry Reid, Chaowei Zhang, Ari Löytynoja, Juha Merilä

**Affiliations:** Area of Ecology and Biodiversity, School of Biological Sciences, The University of Hong Kong, Hong Kong SAR, China; Ecological Genetics Research Unit, Organismal and Evolutionary Biology Research Programme, Faculty of Biological and Environmental Sciences, FI-00014 University of Helsinki, Helsinki, Finland; Institute of Biotechnology, HiLIFE, University of Helsinki, Helsinki, Finland; Area of Ecology and Biodiversity, School of Biological Sciences, The University of Hong Kong, Hong Kong SAR, China; Area of Ecology and Biodiversity, School of Biological Sciences, The University of Hong Kong, Hong Kong SAR, China; Organismal and Evolutionary Biology Research Programme, Faculty of Biological and Environmental Sciences, University of Helsinki, Helsinki, Finland; Area of Ecology and Biodiversity, School of Biological Sciences, The University of Hong Kong, Hong Kong SAR, China; Ecological Genetics Research Unit, Organismal and Evolutionary Biology Research Programme, Faculty of Biological and Environmental Sciences, FI-00014 University of Helsinki, Helsinki, Finland

**Keywords:** isolation, inbreeding, mutation load, purifying selection, *Pungitius pungitius*, runs of homozygosity

## Abstract

The genomic consequences of prolonged population decline and isolation are increasingly recognized, but quantitative assessments of mutation loads have been limited by low population-level replication in individual studies. Moreover, how inbreeding and purifying selection shape the genomic landscape of deleterious variation remains poorly understood. We evaluated the abundance and frequency of putative deleterious mutations, characterized the landscape of deleterious variation, and measured the efficacy of purifying selection in 17 wild nine-spined stickleback (*Pungitius pungitius*) populations covering varying levels of inbreeding (*F*_ROH_ = 0.015 to 0.912) and histories of isolation. We found significantly more deleterious homozygous mutations and a greater frequency of mildly deleterious variants in long-term small, isolated, and inbred populations than in larger outbred populations. Deleterious homozygotes were enriched in runs of homozygosity regions across all study populations, but the extent of enrichment was more pronounced in larger outbred populations than in small inbred populations. Historical effective population sizes serve as an indicator of the strength of purifying selection for mildly deleterious alleles but not for strongly deleterious alleles. The results demonstrate that the accumulation and purging of deleterious variants can occur simultaneously and that a large fraction of segregating strongly deleterious variants are recessive lethals. These findings, which are based on analyses of highly replicated samples of populations, suggest that the level of inbreeding is a good predictor of realized loads of deleterious mutations and that the genomic consequences of prolonged isolation in small populations are predictable.

## Graphical Abstract

**Graphical Abstract:**
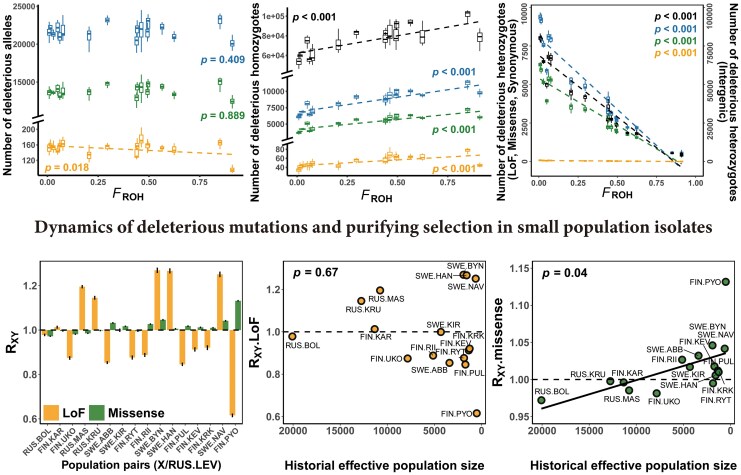

## Introduction

The Anthropocene is characterized by taxonomically widespread and global demographic declines (Finn et al. 2023), along with an increasing number of populations living in patchy and fragmented landscapes. These trends are often driven by the diversification, intensification, and expansion of human activities (Scanes 2018). Consequently, anthropogenic habitat deterioration and fragmentation pose major threats to the long-term viability of many species and populations. These factors not only exacerbate population declines and range contractions (Crooks et al. 2017) but are also accompanied by significant genetic and evolutionary consequences for surviving populations, including increased genetic differentiation and decreased genetic variability and thereby evolvability (Segelbacher et al. 2003; González et al. 2020). Small population isolates are especially at risk of losing genetic variation and a reduced capacity to respond to both stochastic and deterministic environmental changes (Gómez-Fernández et al. 2016). Compared with large and connected populations, they are also more likely to suffer from elevated levels of inbreeding and the accumulation of deleterious mutations, with negative impacts on individual and population fitness (Kyriazis et al. 2023). In general, small population isolates face much higher extinction risks than their large outbred counterparts do (Cowie et al. 2022).

While small isolated populations can survive for hundreds of generations (Dussex et al. 2021), the genomic consequences of prolonged isolation in terms of purging and accumulation of deleterious mutations remain poorly understood. This is due to challenges arising from the time lag between demographic changes and genomic impacts (Dussex et al. 2023), as well as the technical difficulties in accurately identifying truly deleterious mutations and their fitness effects—a central and unresolved question in human genetics (Robinson et al. 2023). Advances in sequencing techniques and computational methods, originally designed for humans and later expanded to a handful of model organisms, have been adapted for broader applications, enabling empirical studies on species of conservation concern, such as Sumatran rhinoceros (*Dicerorhinus sumatrensis*; von Seth et al. 2021), Bengal tigers (*Panthera tigris tigris*; Khan et al. 2021), Iberian lynx (*Lynx pardinus*; Kleinman-Ruiz et al. 2022), alpine ibex (*Capra ibex*; Grossen et al. 2020), island foxes (*Urocyon littoralis*; Robinson et al. 2018), Isle Royale moose (*Alces alces*; Kyriazis et al. 2023), and Kakapo (*Strigops habrotilus*; Dussex et al. 2021), to improve our understanding of the long-term persistence of small populations through the purging of strongly deleterious mutations. However, there is little consensus as to whether a small population size typically increases (Beichman et al. 2019), decreases (Dussex et al. 2021; von Seth et al. 2022), or does not change (Kyriazis et al. 2023) mutation load, which might be influenced by a combination of population demography, evolutionary history, genetic drift, and natural selection (Stoffel et al. 2021).

The interpretations of these heterogeneous patterns are highly variable, ranging from weakened effectiveness of purifying selection in small populations causing the buildup of mutation load (Robinson et al. 2016) to the contrary scenario where enhanced purifying selection has decreased mutation load (Khan et al. 2021). One explanation is that the predicted impact of selection and genetic drift depends on the severity of the consequences of the mutation (Kimura et al. 1963). For example, deleterious mutations, subject to purifying selection, are eliminated from the population (Kimura 1983). The rate of elimination depends on the selection coefficient (*s*) and dominance coefficient (*h*) of the mutation, which reflects its severity relative to the wild-type allele and its impact in the recessive state. If the impact of the mutation is minor or the population is very small (i.e. |*s*| < 1/*N_e_*), selection becomes ineffective, and the allele's frequency fluctuates randomly due to genetic drift (Kimura 1968). Most genetic variability within a species is considered to be neutral, having little or no selective effect, and is shaped primarily by genetic drift (Kimura 1979). Another possible explanation for the heterogeneous outcomes is the small size of empirical studies that have quantified the efficacy or strength of purifying selection (but see Robinson et al. 2022). These studies have typically focused on endangered species and, understandably, consist of at best a few tens of individuals from fewer than a handful of populations. Therefore, empirical case studies based on highly replicated sampling of populations with differing demographic histories and effective population sizes (*N_e_*) are essential for making generalizations and predictions about the genomic consequences of small populations experiencing prolonged isolation. In addition, quantifying the efficacy of purifying selection would help us understand the likelihood of small isolated populations successfully purging their accumulated deleterious mutation loads.

Aside from purifying selection, long-term demographic history has also been found to be a key factor driving patterns of heterozygosity and deleterious variation across the genome (Robinson et al. 2019), but how inbreeding and purifying selection shape the landscape of deleterious variation remains poorly understood. Inbreeding generates homozygous stretches of DNA known as runs of homozygosity (ROH, Broman and Weber 1999), and deleterious alleles can occur either within or outside ROH regions. While studies of humans (Szpiech et al. 2013; Szpiech et al. 2019) and domestic animals (Zhang et al. 2015; Bosse et al. 2019) have shed light on the enrichment of deleterious homozygotes in ROH, especially long ROH regions, it is unclear to what extent this applies to wild populations (but see Humble et al. 2023; Steux and Szpiech 2024) that are solely subject to natural selection. Furthermore, inbreeding levels in human and domestic animal populations are lower (with inbreeding coefficients *F*_ROH_ up to 0.5; but see Bortoluzzi et al. 2020) than those in many small wild populations that have experienced prolonged isolation, such as Brown-eared Pheasants (*Crossoptilon mantchuricum,* 0.6 to 0.8; Wang et al. 2021) and Indian lions (*Panthera leo persica,* 0.8; de Manuel et al. 2020). It is conceivable that differences in inbreeding levels and the strength of natural selection could influence the abundance and distribution of deleterious variants across the genome.

The nine-spined stickleback (*Pungitius pungitius*), a small teleost fish that occurs both in open marine and isolated freshwater habitats, provides an opportunity to study mutation accumulation and purging in a highly replicated design. Different populations of this species vary dramatically in terms of their history of isolation, levels of genetic variability, effective population sizes, and evolutionary history (Merilä 2013; Feng et al. 2022; Kivikoski et al. 2023). Of particular interest are the pond isolates of this species, which show extremely low levels of genetic diversity and high levels of inbreeding (Shikano et al. 2010a; Kivikoski et al. 2023). These populations have been subjected to extremely strong genetic drift; populations separated by just a couple of kilometers can be effectively fixed for different alleles at the majority of their loci (Shikano et al. 2010b; Li et al. 2019).

**Fig. 1.**
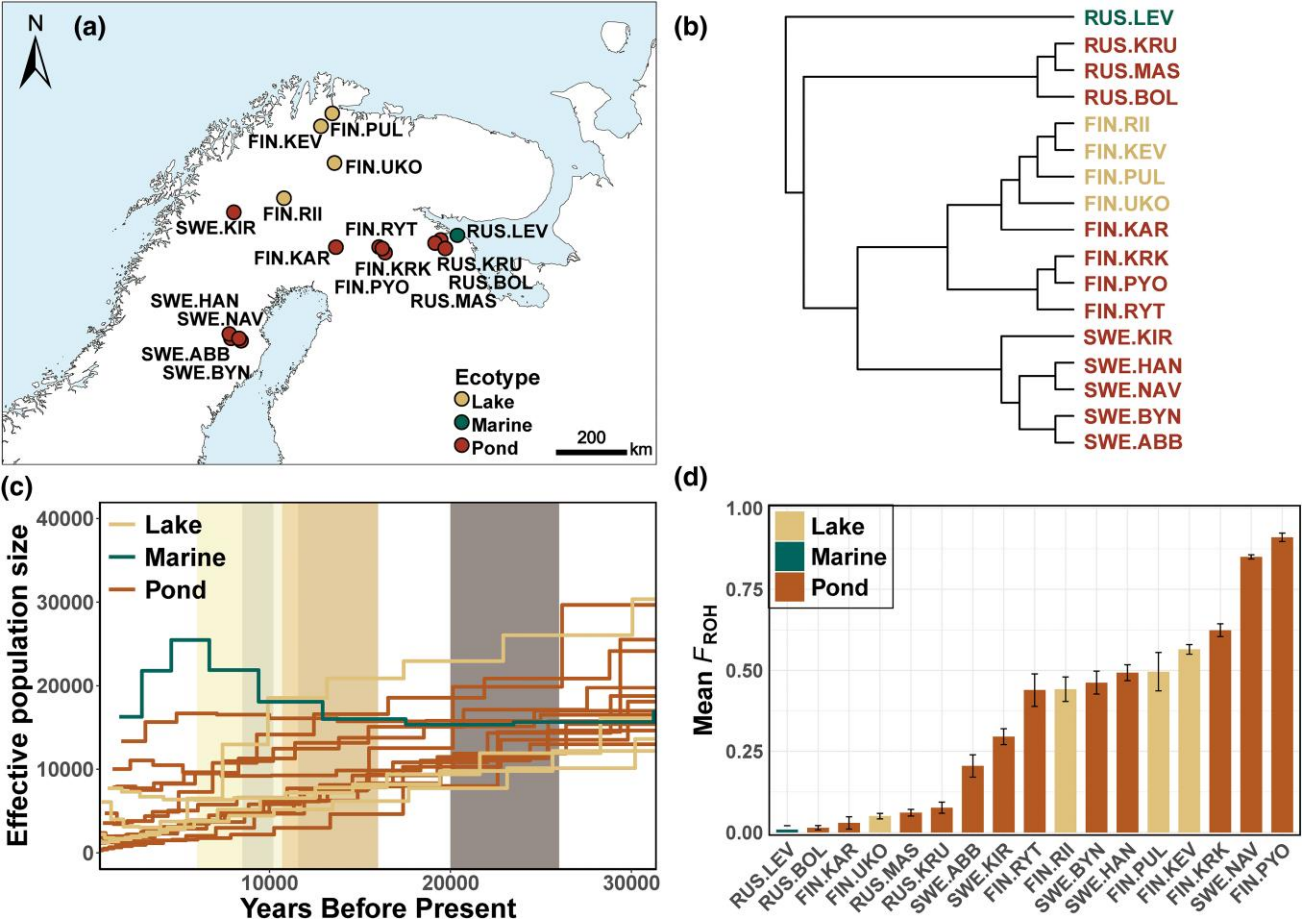
Overview of the 17 nine-spined stickleback (*Pungitius pungitius*) populations, comprising 1 marine population, 4 lake populations, and 12 pond populations. The four subplots are: a) geographical locations of the study populations; b) an ultrametric tree depicting phylogenetic relationships among the populations, derived from Feng et al. (2022); c) demographic history of study populations over the past 30,000 years, derived from Feng et al. (2023). The modern Baltic Sea has evolved over the last four millennia, the yellowish shades represent distinct historical developmental stages of the Baltic Sea, while the gray shading indicates the period of the last glacial maximum (26,000 to 20,000 BC); d) levels of inbreeding, quantified as the proportion of runs of homozygosity regions across the autosomal chromosomes (*F*_ROH_), presented in an ascending order, derived from Kivikoski et al. (2023).

The main purpose of this study was twofold: (i) make generalizations on the genomic mutation loads in small populations under prolonged isolation from highly replicated sampling of populations and (ii) understand how inbreeding shapes the landscape of deleterious variation under natural selection. To this end, we studied 17 nine-spined stickleback populations differing in their levels of inbreeding, ranging from large outbred marine, lake populations to small isolated and inbred pond populations (Fig. 1, supplementary fig. S1, Supplementary Material online). These populations span the extreme ends of the genome-wide ROH distribution in the wild (*F_ROH_* ranging from 1.5% to 91.2%). First, we evaluated the abundance and frequency of putative deleterious alleles, including both strongly and mildly deleterious variants, in individuals with varying levels of inbreeding. Next, we characterized patterns of deleterious variation occurring both outside and inside ROH regions, as well as in different ROH lengths that reflect either historical or recent inbreeding. Finally, we quantified the efficacy of purifying selection against deleterious alleles, homozygotes, and heterozygotes in both large outbred and small inbred populations, along with the efficacy of purging deleterious homozygotes in ROH regions. Ultimately, the results will inform us on the predictability of the genomic consequences of inbreeding, how inbreeding and purifying selection shape the landscape of deleterious variation, whether the consequences differ between historical and recent inbreeding, and whether purging is efficient enough to effectively counteract the accumulation of deleterious variation.

## Results

While the marine population (RUS.LEV) has maintained a roughly stable effective population size (*N_e_*), all freshwater populations have experienced, to varying extents, a gradual decline in their *N_e_* over the past 30,000 years, most pronounced in pond populations (Fig. 1c). Individual inbreeding levels (*F*_ROH_) varied widely, from 0.3% in one marine individual to 92.5% in one pond individual, with an average ROH proportion of 35.8% across all sequenced individuals (*n* = 363; supplementary fig. S1, Supplementary Material online). In freshwater populations, *F*_ROH_ ranged from 1.5% in RUS.BOL to 91.2% in FIN.PYO (Fig. 1d, supplementary table S1, Supplementary Material online).

### Number and Frequency of Deleterious Alleles

**Fig. 2.**
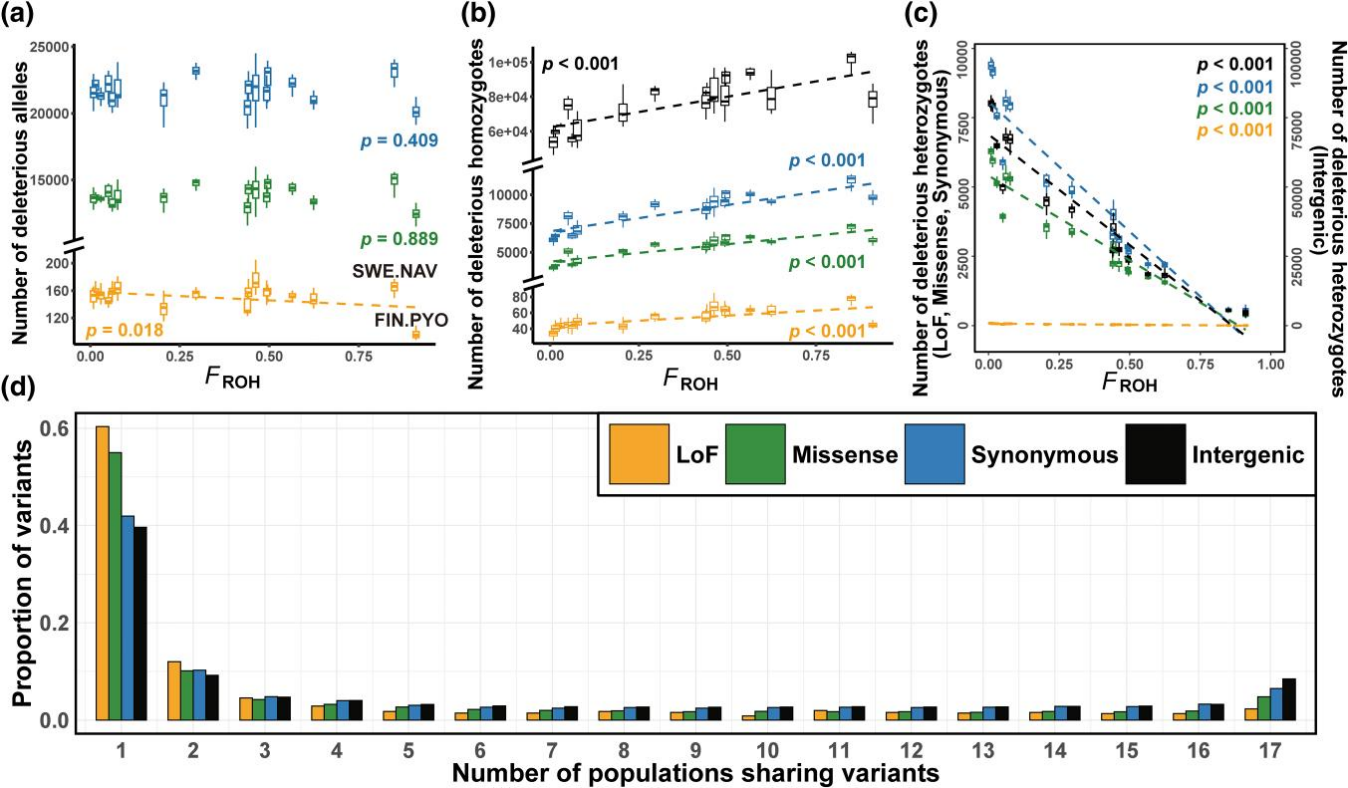
Relationships between inbreeding (*F*_ROH_) and (a) the number of potentially deleterious alleles, b) the number of potentially deleterious homozygotes, and c) heterozygotes for loss-of-function (LoF), missense, synonymous, and intergenic variants. d) Proportion of variants shared by different numbers of populations for LoF, missense, synonymous, and intergenic variants. In subplot (a), a homozygote was counted as having two allele copies, and a heterozygote was counted as having one allele copy. The dashed line in subplots a to c indicated trends that met the significance level of *P* < 0.05. The populations in subplots a to c, listed from left to right, were 1) RUS.LEV, 2) RUS.BOL, 3) FIN.KAR, 4) RUS.MAS, 5) RUS.KRU, 6) FIN.UKO, 7) SWE.ABB, 8) SWE.KIR, 9) FIN.RII, 10) FIN.RYT, 11) SWE.BYN, 12) FIN.PUL, 13) SWE.HAN, 14) FIN.KEV, 15) FIN.KRK, 16) SWE.NAV, and 17) FIN.PYO.

After polarizing and filtering the original 1,825,670 single-nucleotide polymorphism sites, we retained 136,321 variants in protein-coding regions for downstream analyses, including 994 loss-of-function (LoF) variants, 64,746 missense variants, and 70,581 synonymous variants. A total of 562,297 intergenic variants were selected as a neutral baseline for comparison. The total number of missense and synonymous alleles was independent of the level of inbreeding *F*_ROH_, while the number of LoF alleles decreased significantly with increasing *F*_ROH_ (*P* = 0.018, 0.889, 0.409 for LoF, missense, synonymous alleles, respectively, [Fig. 2a, Table 1]). Notably, the number of LoF and missense alleles in the most inbred population, FIN.PYO, was distinctively lower than that in the other populations (Fig. 2a). However, the number of deleterious homozygotes increased, and the number of deleterious heterozygotes decreased with increasing *F*_ROH_ for both LoF, missense and synonymous variants (*P* < 0.001 for all categories, Fig. 2b and c; Table 1). The slope for the increase in LoF and intergenic homozygotes with increasing *F*_ROH_ was shallower than that for missense and synonymous homozygotes (slopes: 1.952, 2.524, 2.647, and 2.670 for LoF, intergenic, missense, and synonymous homozygotes, respectively; Table 1, Fig. 2b). Similarly, the decline in LoF heterozygotes was shallower than that in synonymous heterozygotes (slopes: −2.828, −2.957 for LoF and synonymous heterozygotes; Table 1, Fig. 2c).

**Table 1:** Significance levels derived from generalized linear mixed-effect models measuring the relationship between each response variable and the fraction of the genome in runs of homozygote (ROH) regions

Response variables ∼ Fraction of genome in ROH (*F_ROH_*)	post.mean	Lower 95% CI	Upper 95% CI	pMCMC
Number of deleterious alleles	−70.210	−766.840	644.870	0.884
Number of LoF alleles	−21.159	−37.269	−3.794	0.018
Number of missense alleles	−53.800	−730.900	625.000	0.889
Number of synonymous alleles	−414.000	−1402.600	574.700	0.409
Number of intergenic alleles	−4492.000	−16556.000	9014.000	0.489
Number of deleterious homozygotes	2781.000	2312.000	3192.000	< 0.001
Number of LoF homozygotes	1.952	1.285	2.628	< 0.001
Number of missense homozygotes	2.647	2.253	3.024	< 0.001
Number of synonymous homozygotes	2.670	2.207	3.046	< 0.001
Number of intergenic homozygotes	2.524	2.006	2.991	< 0.001
Number of deleterious heterozygotes	−5588.000	−5866.000	−5317.000	< 0.001
Number of LoF heterozygotes	−2.828	−3.187	−2.431	< 0.001
Number of missense heterozygotes	−2.934	−3.066	−2.770	< 0.001
Number of synonymous heterozygotes	−2.957	−3.102	−2.812	< 0.001
Number of intergenic heterozygotes	−2.939	−3.097	−2.786	< 0.001
Proportion of LoF homozygotes in ROH	−0.405	−1.969	0.967	0.613
Proportion of missense homozygotes in ROH	−0.579	−0.862	−0.324	< 0.001
Proportion of synonymous homozygotes in ROH	−0.571	−0.765	−0.357	< 0.001
Proportion of LoF homozygotes in short ROH	−0.396	−1.816	1.119	0.607
Proportion of missense homozygotes in short ROH	−0.558	−0.850	−0.284	0.002
Proportion of synonymous homozygotes in short ROH	−0.562	−0.796	−0.358	< 0.001
Proportion of LoF homozygotes in long ROH	0.124	−1.612	1.865	0.896
Proportion of missense homozygotes in long ROH	−0.476	−0.984	0.153	0.113
Proportion of synonymous homozygotes in long ROH	−0.415	−1.094	0.265	0.242
LoF/synonymous variants ratio	−0.001	−0.002	0.000	0.056
LoF/synonymous homozygotes ratio	0.000	−0.001	0.001	0.724
LoF/synonymous heterozygotes ratio	0.005	0.004	0.007	< 0.001
Missense/synonymous variants ratio	0.011	0.003	0.018	0.002
Missense/synonymous homozygotes ratio	0.027	0.019	0.036	< 0.001
Missense/synonymous heterozygotes ratio	0.137	0.103	0.168	< 0.001
LoF/synonymous homozygotes in ROH regions	0.000	−0.005	0.005	0.909
LoF/synonymous homozygotes outside ROH regions	0.000	−0.001	0.002	0.593
Missense/synonymous homozygotes in ROH regions	0.019	−0.054	0.100	0.629
Missense/synonymous homozygotes outside ROH regions	0.102	0.086	0.118	< 0.001
LoF/synonymous homozygotes in short ROH	0.001	−0.004	0.006	0.778
LoF/synonymous homozygotes in long ROH	−0.001	−0.019	0.017	0.878
Missense/synonymous homozygotes in short ROH	0.026	−0.049	0.109	0.509
Missense/synonymous homozygotes in long ROH	0.078	−0.290	0.466	0.687

Over half of the LoF and missense variants were unique to individual populations, while most synonymous and intergenic variants were shared across at least two populations (Fig. 2d). Notably, intergenic and synonymous variants were more frequently shared among three or more populations compared to LoF and missense variants, with synonymous variants closely mirroring the distribution of intergenic variants.

### Landscape of Deleterious Homozygotes in and Outside ROH

**Fig. 3.**
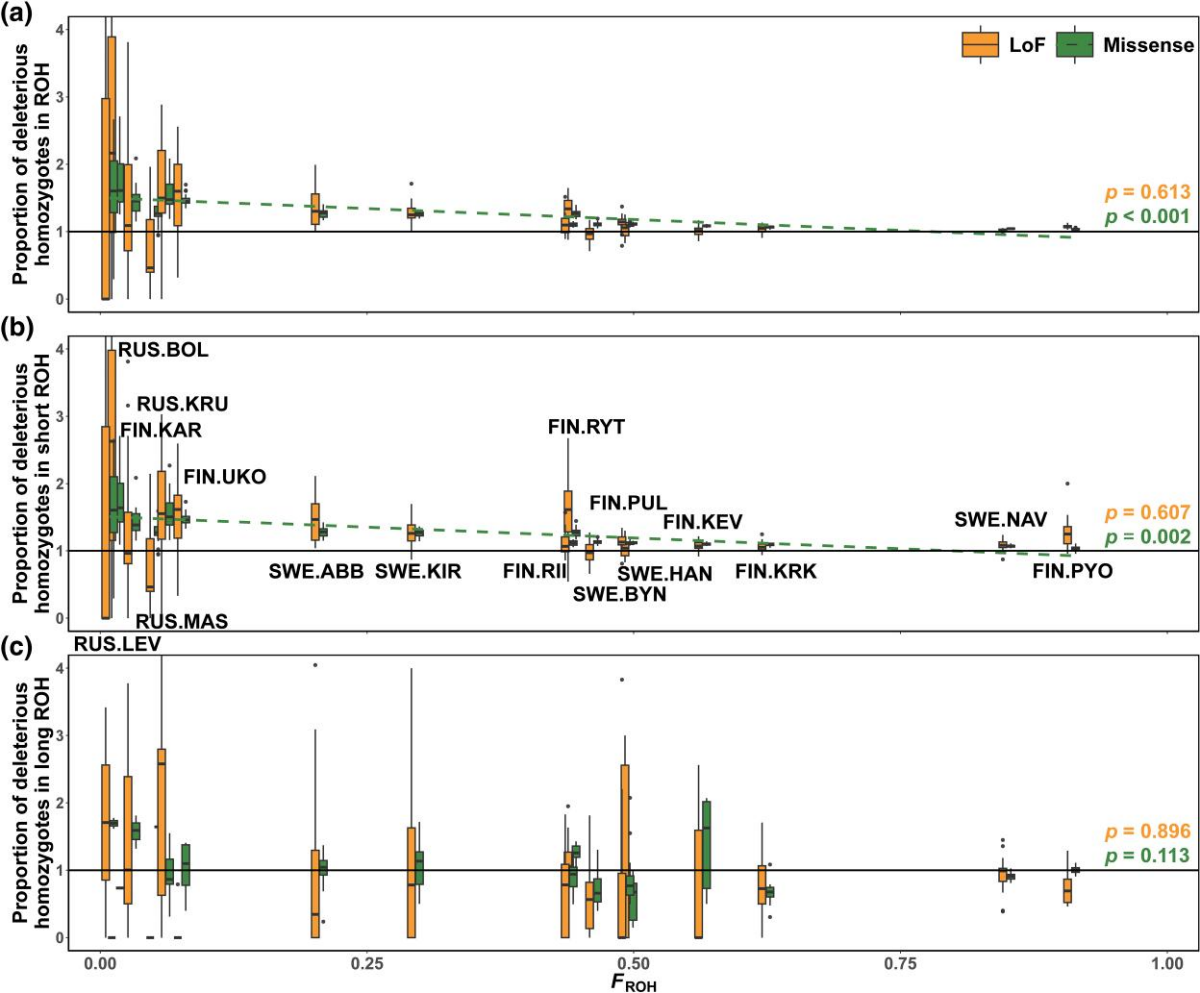
The proportion of deleterious homozygotes falling within a) ROH of any size, b) short ROH regions (100 kb to 1 Mb), and c) long ROH regions (> 2 Mb), normalized by the fraction of the genome covered by ROH of any size, short ROH, or long ROH, respectively, plotted against the level of inbreeding *F*_ROH_ for each population. When the population mean had a proportion of deleterious homozygotes in ROH or short/long ROH, which was higher than one, it indicated an enrichment of deleterious homozygotes in that particular category of ROH. The *P* values were derived from generalized linear mixed-effect models that take into account phylogenetic nonindependence among populations. The dashed line in subplots (a) to (c) indicated trends that met the significance level of *P* < 0.05. The populations, ordered from lowest to highest *F*_ROH_ (from left to right), were RUS.LEV, RUS.BOL, FIN.KAR, RUS.MAS, RUS.KRU, FIN.UKO, SWE.ABB, SWE.KIR, FIN.RII, FIN.RYT, SWE.BYN, FIN.PUL, SWE.HAN, FIN.KEV, FIN.KRK, SWE.NAV, and FIN.PYO.

Both LoF and missense deleterious homozygotes exhibited enrichment in ROH regions, which was a trend observed in both larger outbred and smaller inbred populations ((Fig. 3a, supplementary fig. S2a and b, Supplementary Material online). The partitioning of ROH regions into short and long segments yielded mixed results. While the majority of populations showed enrichment of deleterious homozygotes in short ROH regions (Fig. 3b), this enrichment was not always observed in long ROH regions (Fig. 3c, supplementary fig. S2, Supplementary Material online). For example, the two most inbred populations, SWE.NAV and FIN.PYO, exhibited an excess of deleterious homozygotes in short ROH regions but a deficiency in long ROH regions (Fig. 3b and c).

The extent of deleterious homozygote enrichment varied across populations and differed between LoF and missense alleles. Besides the marine population, which represents a large outbred population (supplementary table S1, Supplementary Material online), we defined large outbred freshwater populations as those with *F*_ROH_ < 0.15 (*n* = 5, including one lake and four large pond populations) and small inbred populations as those with *F_ROH_* > 0.15 (*n* = 11). In large outbred populations, deleterious homozygotes were enriched by up to 180% in ROH, whereas this enrichment decreased abruptly once *F*_ROH_ exceeded 0.55, with only 2% to 8% enrichment in ROH in two most inbred populations (i.e. SWE.NAV, FIN.PYO, Fig. 3a). In particular, the extent of missense homozygote enrichment in ROH or short ROH regions decreased with increasing *F*_ROH_ (Fig. 3a and b, Table 1). Additionally, the proportion of LoF homozygotes in ROHs, whether short or long, was greater than, or similar to, that of missense homozygotes in large outbred populations. However, in small inbred populations, LoF homozygotes were less frequent than missense homozygotes in ROHs, especially in long ROHs.

### Efficacy/Strength of Purifying Selection

**Fig. 4.**
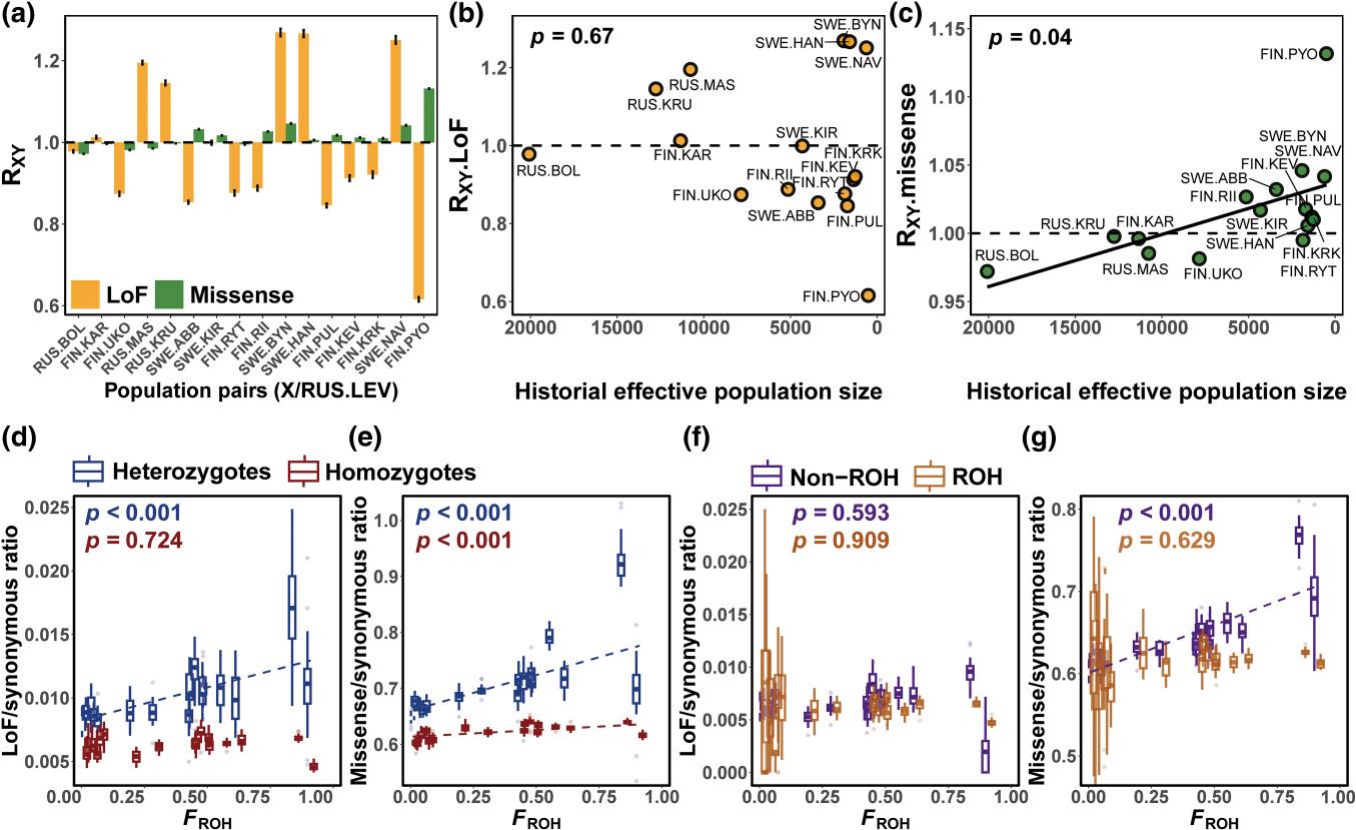
The relative mutational loads (*R_XY_*), a) between each freshwater population and the marine population (RUS.LEV), and the relationship between the *R_XY_* and the historical effective population sizes at around 1000 years before present (derived from Feng et al. (2023) for b) LoF variants and c) missense variants. The error bars of *R_XY_* represent 95% confidence intervals estimated by the jackknifing values across the 20 individual linkage groups, and *R_XY_* indicates deficiency (<1) or excess (>1) of deleterious alleles in a given isolated freshwater population relative to that in an outbred marine population. The efficacy of purifying selection, as indicated by the ratio of the number of deleterious versus synonymous variants, for purging d) LoF and e) missense heterozygous and homozygous alleles, f) LoF homozygous alleles, and g) missense homozygous alleles within ROH and outside ROH against the level of inbreeding *F*_ROH_ for each population. The solid black line in subplot c and the colored dashed lines in subplots d, e, and g indicated trends that met the significance level of *P* < 0.05. The *P* values in subplots (b to g) were derived from generalized linear mixed-effect models that take into account phylogenetic nonindependence among populations. The populations in subplots (d to g), ordered from lowest to highest *F*_ROH_ (from left to right), were RUS.LEV, RUS.BOL, FIN.KAR, RUS.MAS, RUS.KRU, FIN.UKO, SWE.ABB, SWE.KIR, FIN.RII, FIN.RYT, SWE.BYN, FIN.PUL, SWE.HAN, FIN.KEV, FIN.KRK, SWE.NAV, and FIN.PYO.

There were substantial variations among populations in the strength of purifying selection (i.e. *R_XY_*) for LoF alleles, such that neither contemporary nor historical *N_e_* (estimates at 1,000 years before present) served as a reliable indicator of purifying selection against these alleles (*P* = 0.68 and 0.67 for contemporary and historical *N_e_*, respectively [Fig. 4a and b]). Despite both being small and highly inbred, the FIN.PYO population presented a distinct deficiency of LoF alleles compared with the marine population, whereas the SWE.NAV population was in excess (Fig. 4a). However, purifying selection against missense alleles was significantly weakened in small populations, as indicated by larger *R_XY_* values with declining historical *N_e_* (*P* = 0.04, Fig. 4c). The positive association between the strength of purifying selection (*R_XY_*) against mildly deleterious variants and *N_e_* holds true only for historical *N_e_*, but not for contemporary *N_e_* (*P* = 0.30, supplementary fig. S3, Supplementary Material online). To validate the observed patterns and rule out artifacts of genome-wide heterogeneity, we calculated *R_XY_* via variants located within highly conserved single-copy benchmarking universal single-copy ortholog (BUSCO) genes. This relationship between *R_XY.BUSCO_* and historical *N_e_* remained consistent at the LoF and missense sites (supplementary fig. S3, Supplementary Material online).

The relative proportion of LoF alleles did not increase with increasing levels of inbreeding (*P* = 0.056), whereas the proportion of missense alleles did (*P* = 0.002, Table 1). Specifically, the relative increase in missense heterozygous alleles with increasing *F*_ROH_ was more pronounced than that of homozygous alleles and, to a lesser extent, LoF heterozygous alleles (slope of 0.137, 0.027, and 0.005 for missense heterozygotes, missense homozygotes, and LoF heterozygotes, respectively, Fig. 4d and e, Table 1). This suggests that a substantial fraction of amino acid-altering mutations are recessive or nearly recessive, experiencing weaker selection when they are in a heterozygous state. Notably, a strong signal of purging deleterious homozygotes was observed in the most inbred population, FIN.PYO (Fig. 4d and e). Additionally, with the increase of inbreeding levels, there was no significant change in the relative proportion of LoF homozygotes within ROH or non-ROH regions (*P* = 0.909 and 0.593 respectively), regardless of whether the ROH regions were short or long (*P* = 0.778, 0.878, respectively, Fig. 4f, supplementary fig. S4, Supplementary Material online, Table 1). However, the relative proportion of missense homozygotes outside ROH regions increased with rising *F*_ROH_ (*P* < 0.001, Fig. 4g).

## Discussion

Due to the complexity and stochasticity of biological processes influencing the dynamics of mutational loads, the genomic consequences of prolonged isolation are still not fully understood (Glémin 2003; Robinson et al. 2023). To the best of our knowledge, the present study represents the most replicated empirical study that quantifies loads of (putatively) deleterious mutations in the wild and serves as one of the few studies that have quantified the dynamics of purifying selection acting on them. The most salient findings of this study include the following: (i) despite an increase in the absolute number of deleterious homozygotes with increasing *F*_ROH_, the extent of increase for strongly deleterious homozygotes was less pronounced than that for neutral variants (Fig. 2b); (ii) deleterious homozygotes were enriched in ROH regions across all study populations with *F*_ROH_ ranging from low (0.02) to high (0.91), and the extent of enrichment was more pronounced in large outbred populations than in small inbred populations (Fig. 3a); (iii) while historical inbreeding has led to the accumulation of deleterious variants, recent inbreeding has facilitated the purging of these variants (Fig. 3b and c); (iv) historical effective population sizes served as an indicator of the intensity of purifying selection against mildly deleterious alleles but not against strongly deleterious alleles (Fig. 4b and c); and (v) purifying selection was more strongly weakened against deleterious heterozygous alleles than against homozygous alleles, with the difference being more pronounced in small inbred populations (Fig. 4d and e); and 6) although purging strongly deleterious homozygous alleles remained effective in small inbred populations compared to large outbred ones, and it was insufficient to prevent a significant increase in their homozygous mutation loads (Figs. 2b and 4b).

Our results provide clear and strong evidence of the negative genomic consequences of prolonged isolation in small populations. While previous studies have reported contradictory trends, such as increased (Beichman et al. 2019), decreased (Mathur et al. 2023), or unchanged numbers or frequencies of deleterious alleles (Kyriazis et al. 2023) in small populations compared with large populations, we observed significant variation in the total number of deleterious alleles across populations, encompassing all of these scenarios. Overall, the total number of mildly deleterious alleles did not distinctly increase with ascending *F*_ROH_, whereas strongly deleterious alleles declined with increasing *F*_ROH_. This decline was primarily driven by the low number of LoF alleles in the most inbred population, FIN.PYO. However, we observed a notable increase in the fixed mutational load and relatively high frequency of mildly deleterious mutations across many small populations compared with the marine population. Hence, a slow fitness decline and further decrease in population size can be expected along with the continuous fixation of deleterious mutations, reducing the viability of the study populations over the long term. This is especially true as these populations are facing changing environmental conditions due to a warming climate (Kivinen et al. 2017), and consequently; they are likely to face increased selective pressure to cope with these changing conditions.

Given that effective population size determines the amount of genetic drift a population experiences (Wright 1931) and that drift, in turn, increases the fixation probability of deleterious alleles (Kimura 1968), it was not surprising that the number of deleterious homozygotes was a strong positive function of the level of inbreeding. This positive association has also been observed in Bengal tigers (Khan et al. 2021), Chinese crocodile lizards (*Shinisaurus crocodilurus*; Xie et al. 2022), and montane red foxes (*Vulpes vulpes*; Quinn et al. 2024), although with fewer individuals in three to four populations. Hence, these findings strongly support the notion that a loss of genetic variation, coupled with a declining population size, is accompanied by the accumulation of homozygous deleterious variants. An increased number or proportion of deleterious homozygotes in small populations has been noted in many case studies, including but not limited to three-spined sticklebacks: *Gasterosteus aculeatus* (Yoshida et al. 2020), eastern massasauga rattlesnakes *Sistrurus catenatus* (Ochoa and Gibbs 2021), Iberian lynx (Kleinman-Ruiz et al. 2022), and the Apennine brown bear *Ursus arctos marsicanus* (Benazzo et al. 2017). Since the homozygous mutation load can be considered a proxy of inbreeding depression (Charlesworth and Charlesworth 1987), it can limit the recovery of endangered populations by reducing their fitness. In the context of nine-spined sticklebacks, elevated levels of fluctuating asymmetry—a measure of developmental stability (Graham et al. 2010)—have been observed in isolated pond populations (Trokovic et al. 2012). When we correlated the levels of fluctuating asymmetry with *F*_ROH_ for the five populations included in our study, there was a strong positive correlation (*r_s_* = 0.9, *n* = 5, *P* = 0.04), suggesting an association between the inbreeding level and developmental stability (supplementary fig. S5, Supplementary Material online). This suggests that there is a fitness decline as a result of inbreeding depression in these populations, but further study is needed to confirm this finding, with a focus on actual fitness components.

As expected from theoretical grounds (Kimura 1983; Ohta and Tachida 1990) and previous empirical studies (Robinson et al. 2022), more heterozygous deleterious variants were observed in larger populations with higher genome-wide heterozygosity than in smaller populations with lower heterozygosity. In contrast to homozygous deleterious alleles, which likely affect individual fitness in the present generation (Charlesworth and Charlesworth 1987), heterozygous deleterious alleles can reduce individual fitness in future generations, for example, if the population becomes more inbred (Bertorelle et al. 2022). Deleterious mutations can accumulate in large populations as recessive (or partially recessive) heterozygotes hidden from natural selection, especially those with small to moderate effects, making purging unlikely to be efficient against them (Kimura 1968; Ohta 1973). In small, isolated populations, deleterious heterozygous mutations can be reduced after a prolonged bottleneck (Nei et al. 1975; Glémin 2003) or converted to homozygotes through inbreeding and genetic drift (Kimura 1968). In line with this, the number of missense and LoF heterozygotes was a negative function of *F*_ROH_ level, whereas the number of homozygotes increased with increasing *F*_ROH_ level (Fig. 2b and c).

We observed that ROH regions harbor proportionally more deleterious homozygotes than the rest of the genome does. This finding agrees with observations from human (Szpiech et al. 2013; Mezzavilla et al. 2015; Szpiech et al. 2019), domestic (Marsden et al. 2016; Bosse et al. 2019; Bortoluzzi et al. 2020), and wild animal populations (Humble et al. 2023; Mathur et al. 2023; Steux and Szpiech 2024). The enrichment of deleterious mutations in ROH regions stands firmly in our study populations even at the extreme ends of ROH coverage (cf. 1.5% and 91.2% of the genome in ROH). However, the enrichment is clearly more pronounced in large outbred populations, indicating that in these populations, deleterious mutations become homozygous owing to ROHs created by recent inbreeding. In contrast, in most inbred populations, the genome is nearly completely homozygous, making marked enrichment within ROH regions impossible. Our observation of a greater proportion of deleterious homozygotes in ROHs in large populations challenges the common perception that ROH regions primarily create deleterious homozygotes in small inbred populations.

In humans, domestic animals, and wild macaques, long ROH regions accumulate deleterious mutations (Szpiech et al. 2013; Bosse et al. 2019). Conversely, we found that, in small, inbred stickleback populations, strongly deleterious homozygotes in long ROH appeared to be purged, as indicated by the lack of increase in the proportion of LoF homozygotes with increasing *F*_ROH_. Over half (*n* = 9) of our study populations are highly inbred (*F*_ROH_ > 0.4), a level of inbreeding that has not been covered by similar studies in humans, domestic animals or wild populations. In more inbred populations, where haplotypes are almost identical and recombination is barely observable, ROH length may be less indicative of ROH age. Consequently, long ROH in these populations might not necessarily originate from recent inbreeding. This allows purifying selection to continuously eliminate strongly deleterious homozygotes within long ROH, thereby reducing their total number, and the remaining LoF variants might be more benign. There was a small proportion of LoF variants (14%) shared in more than half of the study populations (Fig. 2d), implying they were likely not lethal, as otherwise they would not have been maintained. On the other hand, the elimination of a substantial proportion of strongly deleterious homozygotes in long ROH might have enabled these highly inbred populations to survive.

Natural selection continuously removes deleterious mutations, leading to older and shorter ROH with fewer mutations (Stoffel et al. 2021). For example, deleterious mutations are less common in short ROHs than are tolerated mutations in wild populations of Montezuma Quail *Cyrtonyx montezumae* (Mathur et al. 2023). However, we found that deleterious mutations, especially mildly deleterious homozygotes, accumulated in short ROHs. This might reflect the inefficiency of purifying selection in eliminating all deleterious mutations, leading to increased mutation loads in older haplotypes with shorter ROH, as has often been observed in humans and domestic animals. On the other hand, while most freshwater populations have evolved independently, each accumulating their own distinct mutation load (Fig. 2d), the small proportion of LoF and missense variants shared across all study populations indicated these were likely inherited from ancestral populations and retained within short ROHs. Additionally, we cannot rule out the possibility that deleterious alleles can hitchhike with positively selected beneficial variants to be retained: an explanation evoked to explain the enrichment of deleterious mutations in short ROH regions in cattle (Zhang et al. 2015). Hence, these mixed results from human, domesticated, and wild populations indicate diversification in the mutation load dynamics of ROH regions.

Quantitative data on the efficiency of purifying selection from the wild are rare, and there is considerable variation in qualitative interpretations ranging from strong purging (e.g. Beichman et al. 2019; Khan et al. 2021; Kleinman-Ruiz et al. 2022) to apparent relaxation of purifying selection (Robinson et al. 2016; Wang et al. 2021) from studies of small wild populations of birds, reptiles, and mammals. While theory predicts that purifying selection is less effective in small populations than in large populations (Kimura 1983; Ohta 1992), empirical data supporting this theory are scarce. Our findings indicate that while historical effective population size can predict the intensity of purifying selection against mildly deleterious alleles, this predictability does not extend to strongly deleterious alleles. There was a large variation in the degree to which strongly deleterious alleles were purged in pond populations (Fig. 4b), likely because of the strong genetic drift that these populations have been subjected to (Shikano et al. 2010a). These patterns aligned with contrasting examples from the wild: some small populations, such as the island fox (Robinson et al. 2018), have successfully purged lethal deleterious alleles and persist, while others, like the extinct mainland kakapo (Dussex et al. 2021), failed to do so and faced extinction. However, our gene annotation is based on an FIN.PYO individual, and we cannot exclude the possibility that the gene or exon set has evolved in more distantly related populations and that the annotated LoF variants do not always lead to total loss of function. Yet, the elevated number of fixed LoF mutations in the majority of small inbred populations shows that purging has not been effective enough to prevent the accumulation of fixed mutation loads in stickleback populations.

The role of demographic history in shaping mutation load and purging remains unclear, with studies reporting both significant impacts (Robinson et al. 2019) and limited effects (Kleinman-Ruiz et al. 2022). This inconsistency likely stems from the dynamic interplay between demographic history and purging processes. Consequently, linking contemporary mutation load estimates with snapshot measures of effective population size across different time periods may not always yield consistent results. Our study revealed that historical, rather than contemporary, effective population sizes drive the purging of mildly deleterious alleles, as purging requires time to manifest. Conversely, continuous population decline amplifies genetic drift, potentially counteracting natural selection and weakening the relationship between contemporary population size and purging.

The anticipated effects of selection and genetic drift are influenced by the severity of the mutation's impact (Kimura 1979). We observed a less pronounced increase in strongly deleterious homozygous alleles with increasing *F*_ROH_ than in neutral alleles, and the decrease in the number of strongly deleterious heterozygotes was also less pronounced. This finding indicates that purifying selection was more efficient at removing strongly deleterious homozygous alleles but less effective at eliminating mildly deleterious heterozygous alleles in small inbred populations. This was evidenced by the greater increase in the ratio of deleterious to synonymous heterozygotes than in homozygotes with increasing *F*_ROH_. While we could not differentiate between additive and recessive deleterious alleles, the aforementioned finding indicates that deleterious mutations are largely recessive and can evade natural selection when heterozygous (Charlesworth and Charlesworth 1999). While recessive lethal alleles can be efficiently purged in small populations where they easily become homozygous, harmful but not lethal alleles can increase in frequency due to drift in small populations (Wright 1931; Ohta 1973). Notably, missense homozygotes in non-ROH regions in small inbred stickleback populations were less likely to be purged than those in ROH regions were, indicating genomic heterogeneity in the action of natural selection. This also suggests that some of the remaining non-ROH regions in the genomes of the most inbred individuals are created by deleterious missense mutations: as individuals with lethal homozygous variants die before reaching maturity (and thus before sampling), only heterozygous haplotypes, defining non-ROH regions, are observed.

The most inbred population in our study, FIN.PYO exhibited a genome-wide *F_ROH_* of 0.912. As a highly inbred population, FIN.PYO serves as an excellent example of how the accumulation and purging of deleterious variants can occur simultaneously. Along with the SWE.NAV population, FIN.PYO has the highest number of homozygous derived mutations, and according to *R_XY_*, purifying selection has been ineffective in removing missense mutations, allowing harmful but nonlethal mutations to accumulate in the genome. However, the *R_XY_* for LoF variants is strongly negative, indicating efficient purging of strongly harmful mutations. Hence, our results demonstrate the purging of deleterious mutations in action and indicate that a large fraction of LoF variants segregated in FIN.PYO are recessive lethals that are tolerated as heterozygotes but are unviable as homozygotes (Fig. 4d).

While it would be both interesting and valuable to include more populations from Western European lineage or introgressed populations (cf. Feng et al. 2022) to better understand the impacts of population demography, genetic drift, and natural selection on mutation load, our data presents significant challenges for such an analysis. First, when migration occurs in the form of admixture, some alternative alleles may reflect differential adaptation in ancestral populations and should not be classified as part of the negative load. Second, there is a practical limitation: we have sampled a large number of freshwater populations from the Eastern European lineage, whereas most admixed or Western European lineage populations are only available from marine environments (Feng et al. 2022). Despite these challenges, we encourage future studies to address these limitations in order to investigate the impacts of migration on mutation load by including these populations.

The importance of genetic studies in the recovery of endangered populations has long been recognized (Madsen et al. 1999). While our study cannot provide a definitive answer on the best candidate population for a genetic rescue program, it highlights that the optimal approach depends on the specific circumstances. We found that the number of derived deleterious mutations is relatively consistent across populations of different sizes, but in small populations, these mutations are more often homozygous or even fixed. If individuals from a small population are used for genetic rescue, they probably carry such fixed deleterious mutations and may have lower fitness than individuals from a larger population do. Conversely, while homozygous mutations in these individuals may be harmful, they are evidently not lethal; otherwise, the individuals would not survive to be observed. In the case of Isle Royale gray wolves, recessive lethal mutations hiding in the large outbred population became homozygous in the tiny island population and ultimately caused its demise (Robinson et al. 2019). If the endangered population is not in such an extreme situation, there is even hope for population growth when genetic rescue candidates come from a large population, as they introduce the smallest amount of fixed deleterious variation. However, lethal variation is most efficiently purged in small populations, which may also represent a more diverse reservoir of adaptive genetic variation (Willi et al. 2007). Therefore, although not ideal, individuals from another small population might be a better solution for the genetic rescue of very small populations.

## Conclusions

The heterogeneity of patterns observed in the literature inspired this study to quantify deleterious mutation loads and the efficacy of purging in relation to levels of inbreeding and effective population size. Using a large sample of populations covering an exceptionally broad range of inbreeding levels, this study breaks through limitations of existing studies, which have focused primarily on endangered species with limited replication at the population level. The results highlight that the consequences of inbreeding for loads of mildly deleterious protein-coding mutations are highly predictable: increasing levels of inbreeding lead to increased loads of deleterious variants. While purging strongly deleterious variants remained efficient even in highly inbred populations (with some notable exceptions), purging mildly deleterious variants was more efficient in historically large populations subject to reduced genetic drift. However, although purging occurred, it was not sufficient to counteract the accumulation of deleterious mutation loads. In other words, while purging has helped reduce the load of deleterious mutations, the rate at which this has happened has not been adequate to prevent their accumulation. In general, the results provide a rare quantitative glimpse of how the interplay between inbreeding and purging determines the loads of deleterious mutations in the wild. Further studies should seek to elucidate the actual fitness consequences of the observed mutation loads. Our exploratory analysis indicates that levels of fluctuating asymmetry increase with increasing levels of inbreeding, along with evidence that inbreeding in this species leads to reduced fitness (Fraimout et al. 2023), suggesting that most inbred populations studied here might suffer from markedly reduced fitness. If so, this raises concerns about their long-term viability, especially given that increasing levels of environmental stress (such as those caused by climate change) are known to amplify the negative effects of inbreeding (Bijlsma and Putten 1999; Kristensen et al. 2003).

## Materials and Methods

### Study Populations and Data Processing

As a model system that has been extensively studied for nearly three decades, the nine-spined stickleback boasts a chromosome-level reference genome assembly of high quality with 25,062 annotated protein-coding genes (Varadharajan et al. 2019; Kivikoski et al. 2021; Wang et al. 2024). We utilized whole-genome resequencing data from 363 individuals from 17 nine-spined stickleback populations, including one marine and 16 freshwater (12 pond, four lake) populations from Russia, Finland, and Sweden (Fig. 1, supplementary table S1, Supplementary Material online; Feng et al. 2022). These populations belong to the Eastern European lineage (EL) and exhibit minimal to no introgression from their Western European lineage (WL) counterparts (Feng et al. 2022; Kivikoski et al. 2023; Yi et al. 2024). The dataset is part of a larger dataset of 889 individuals from 45 populations used by Feng et al. (2022) and Kivikoski et al. (2023), where more details can be found regarding sample collection and data processing. We excluded 25 populations from these previous studies because they either represent divergent WL populations or are EL populations introgressed with WL variants (Feng et al. 2022). We focused exclusively on EL-origin populations, all of which are derived from an ancestral population in the White Sea area following the Last Glacial Maximum.

In brief, whole-genome sequencing at depths of 10 to 20× (supplementary fig. S6, Supplementary Material online) was performed via Illumina short-read (150-bp) paired-end protocols (HiSeq 2500/4000 instruments) at the Beijing Genomics Institute (Hong Kong SAR, China), Novogene Company Limited (UK), and the DNA Sequencing and Genomics Laboratory at the University of Helsinki (Helsinki, Finland). The short-read data were mapped to the published ver. 7 nine-spined stickleback reference genome (Kivikoski et al. 2021) on the basis of a male individual from a Finnish pond population (i.e. FIN.PYO), and variant calling was performed with the Genome Analysis Toolkit (GATK) v.4.0.1.2 following GATK Best Practices workflows (McKenna et al. 2010). Single-nucleotide polymorphisms (SNPs) were extracted using bcftools v.1.7 (Danecek et al. 2021), with which the protein-coding regions of the genome were annotated using SnpEff v4.3 (Cingolani et al. 2012). Variants were filtered out using vcftools v.0.1.5 (Danecek et al. 2011) and bcftools v.1.7 (Danecek et al. 2021) if they were located within repetitive sequences (Varadharajan et al. 2019), unmappable regions, negative mappability mask regions (Kivikoski et al. 2021), with too low or high average coverage (either <8× or >25×), low genotype quality (<20), low quality scores (<30), and more than 20% missing data. We also excluded SNPs with annotation warnings, indicating possible inaccuracies in variant annotation and those located on the known sex chromosomes (LG12) of the EL (Natri et al. 2019).

### Estimating the Levels of Inbreeding

To estimate the levels of inbreeding, we called and analyzed the runs of homozygosity (ROHs) from the genomic data following (Kivikoski et al. 2023). ROHs are uninterrupted long stretches of homozygotes as a result of population size reduction, consanguinity, and natural selection (Szpiech et al. 2013). We used BCFtools roh v.1.16 (Danecek et al. 2021) to calculate runs of homozygosity with the following parameters: -G 30 -I, to account for genotype error, and provided the nine-spined stickleback linkage map (-m flag, Kivikoski et al. 2023) to incorporate accurate recombination rates. We selected the full dataset i.e. SNPs across the entire genome, for ROH calculation, rather than limiting analysis to SNPs within positively masked regions, for several key considerations. The positive mask targets two types of errors: (i) erroneous variants caused by mismapping of reads across repetitive sequence regions, and (ii) lack of variants due to zero genotype qualities resulting from ambiguous read mapping. The first type of error is expected to reduce the length of ROHs, while the second type is expected to increase them. If using positive masked data in the ROH analysis, we can avoid the first error but not the second, potentially leading to an overestimation of ROH lengths. When using the full dataset, both types of errors were retained, and more accurate inferences are expected. Based on our results, these errors are not substantial, and we observe both extremes of the scale.

Regarding ROH filtering, we only applied a minimum length (100 kb) filter, avoiding additional SNP-based thresholds. This length threshold has been adopted widely in multiple cases of birds, reptiles, mammals, or humans (e.g. Kirin et al. 2010; Khan et al. 2021; Ochoa and Gibbs 2021). Given the significant variation in genetic diversity across our 17 study populations (15-fold variation, Kivikoski et al. 2023), applying filters based on metrics such as the number of SNPs was not straightforward. For example, the average inter-SNP distance in RUS.BOL and FIN.PYO was approximately 371 bp and 11,000 bp, respectively. This means that a 100-kb ROH would be supported by ∼269 polymorphic SNPs in RUS-BOL but only 9 SNPs in FIN-PYO.

*F*_ROH_ was calculated as the fraction of the autosomal genome in ROH over 100 kb. Long ROHs that have not been broken by recombination are likely the result of recent inbreeding (McQuillan et al. 2008), whereas short ROHs reflect historical inbreeding (Kirin et al. 2010) or shared ancestry. We calculated *F*_ROH_ for three ROH length (*L*) classes, which were defined as short (100 kb < *L* < 1 Mb), medium (1 Mb < *L* ≤ 2 Mb), and long (2 Mb < *L*), to estimate inbreeding arising from ancestors in different historical time periods. The coalescent times of ROHs can be estimated using the formula *g* = 100/(2*rL*) (Thompson 2013), where “*g*” represents the expected time (in generations) back to the common ancestor when the identical-by-descent haplotypes forming an ROH coalesce, and “*r*” is the recombination rate, which for nine-spined stickleback, has been estimated to be 4.10 cM/Mb (Kivikoski et al. 2021). Therefore, historical inbreeding was estimated at > 122 generations, and recent inbreeding was estimated at < 6 generations.

### Identification and Classification of Putative Deleterious Variants

We polarized all protein-coding variants to ancestral or derived states, using one individual from an ancestral marine population in Japan as an outgroup (supplementary table S1, Supplementary Material online). We eliminated heterozygous sites in the outgroup because of their indeterminable ancestral state. If the outgroup genotype was 0/0, the reference (REF) allele was considered the ancestral state, and no modification was needed. If the outgroup genotype was 1/1, the reference and alternative (ALT) alleles were swapped, and all sample genotypes were reversed. Next, we used the repolarized reference alleles to create an updated reference genome with GATK *FastaAlternateReferenceMaker*, which was then used for variant annotation with SnpEff (Cingolani et al. 2012).

We identified putative deleterious variants as alleles derived from the ancestral form and categorized them into two groups, strongly and mildly deleterious variants, on the basis of their predicted impacts on the encoded amino acids (SnpEff, Cingolani et al. 2012): (1) strongly deleterious variants: loss-of-function (LoF) variants, including stop codons, splice donors or acceptors, and start codon lost variants (supplementary table S2, Supplementary Material online). These variants are assumed to have a disruptive impact on proteins, probably causing protein truncation or triggering nonsense-mediated decay, (2) mildly deleterious variants: missense variants that are nondisruptive and might affect protein effectiveness, and (3) slightly deleterious variants: synonymous variants that are less likely to change protein behavior. Moreover, we retained intergenic variants as a baseline to represent neutral variants. We calculated the proportion of putative deleterious and intergenic variants shared across varying numbers of populations, ranging from none to all. Owing to the challenges in knowing the actual fitness effects of these mutations, they are, strictly speaking, putative deleterious mutations. However, estimates at the individual level can be used as proxies for the impact on relative fitness (Dussex et al. 2023), as the derived counts in individuals provide an appropriate and robust measure of the deleterious burden that can be interpreted at the population level (Robinson et al. 2023). For the sake of brevity, they will be referred to as deleterious variants throughout the text.

### Quantification of Mutational Load

The mutational load, typically defined as the reduction in mean fitness in a population caused by deleterious variation relative to a mutation-free population (Kimura et al. 1963), was quantified in three different ways: the total number of deleterious alleles, the number of deleterious homozygotes, and the number of deleterious heterozygotes, which were specifically measured for LoF, missense and synonymous variants. For the total number of deleterious alleles, a homozygote was counted as two alleles, and a heterozygote was counted as one allele. We also calculated the proportion of homozygous alleles in ROHs, long or short ROHs, for both strongly and mildly deleterious variants. This proportion was normalized to the fraction of the genome covered by ROHs of any size, i.e. long or short ROHs. For population-level estimates, the number of deleterious homozygotes per individual within ROHs or non-ROHs, and within long or short ROHs, was normalized by the corresponding length of the ROH or non-ROH, long or short ROH to eliminate any bias introduced by length variation.

### Efficacy of Purifying Selection

To estimate the relative excess of deleterious variants (including LoF and missense mutations) in freshwater populations with respect to the outbred marine population, we calculated the *R_XY_* statistics between each freshwater population and a marine population (RUS.LEV) using all protein-coding variants (Xue et al. 2015). *R_XY_* is a population-specific statistical estimate of mutational load that measures the relative frequency of a mutation between a pair of populations by comparing the deleterious allele frequencies relative to the frequencies of neutral alleles (Xue et al. 2015). The same number of variants as for deleterious variants was randomly selected from the intergenic regions to serve as a proxy for the neutral level of genetic variation. We calculated the 95% confidence intervals for *R_XY_* based on jackknife estimates across the 20 autosomal linkage groups separately (von Seth et al. 2022). When *R_XY_* > 1 or *R_XY_* < 1, it indicates an excess or deficiency of the focal alleles, respectively, in the freshwater population compared with the marine population, and the latter case implies the presence of purifying selection. We also calculated *R_XY_* using variants located within the 3,209 single-copy BUSCO genes (based on BUSCO Actinopterygii odb9, Simão et al. 2015) to verify the observed patterns. Additionally, we calculated the ratio of the number of putatively deleterious variants (i.e. LoF, missense) to the number of synonymous variants as a proxy for the efficacy of purifying selection (Robinson et al. 2022). This ratio was quantified at both the individual and population levels.

### Statistical Analyses

Relationships between inbreeding (*F*_ROH_) and each response variable were established by fitting generalized linear mixed-effects models using the MCMCglmm package v.2.34 (Hadfield 2010) in R v.4.2.2 (R Development 2022). These response variables included those that quantify the levels of mutation load (e.g. the total number of derived deleterious alleles, the number and the proportion of homozygous or heterozygous deleterious alleles) and the efficacy of purifying selection (see details in Table 1). To compare the extent of increase or decrease among LoF, missense, synonymous, intergenic homozygotes or heterozygotes with increasing *F*_ROH_, we standardized the response variables by subtracting the mean and dividing by the standard deviation. To account for phylogenetic nonindependence (Stone et al. 2011), a phylogenetic correlation matrix was provided as a random effect. This matrix was obtained in PLINK v.1.90 (Purcell et al. 2007) by calculating the identity-by-state distance of all individuals using VCF files of the longest autosome (LG4, Kivikoski et al. 2021). The rooted neighbor-joining tree was then built through the R package ape v.5.6 (Paradis and Schliep 2019) using *Pungitius tymensis* from Hokkaido, Japan (43°49′40″N, 145°5′10″E, Feng et al. 2022), as an outgroup. The tree was converted to a phylogenetic variance–covariance matrix (Kivikoski et al. 2023), and the models were fitted with the uninformative prior: list (*R* = list(*V* = 1, nu = 0.002), *G* = list(G1 = list(V = 1, nu = 1, alpha.mu = 0, alpha. V = 1000))). We configured the parameters as follows: nitt = 100,000 (total interations), burnin = 10,000 (initial iterations discarded for convergence), and thin = 10 (storing every tenth iteration in memory. To test whether smaller populations tend to harbor a relative excess of deleterious variants, we built generalized linear mixed-effects models associating *R_XY_* with either the contemporary (i.e. estimated by program CurrentNe2; available at https://github.com/esrud/currentne2) or historical effective population sizes (*N_e,_* point estimates at 1,000 years before present by program MSMC2, Schiffels and Wang 2020), both derived from Feng et al. (2023). This model accounted for phylogenetic nonindependence among populations by incorporating a phylogenetic correlation matrix as a random effect, which included one randomly sampled individual per population.

## Supplementary Material

msaf110_Supplementary_Data

## Data Availability

The whole-genome resequencing data of wild-caught individuals have been published previously by Feng et al. (2022) and can be accessed on the European Nucleotide Archive (https://www.ebi.ac.uk/ena) under accession codes PRJEB39599 and Zenodo Open Repository: https://zenodo.org/record/6951309, respectively. The bioinformatic codes and scripts used in this study are available from the Zenodo Open Repository (https://zenodo.org/records/15361784).
