# Supplementary material for: Dynamics of Deleterious Mutations and Purifying Selection in Small Population Isolates
Source: Mol Biol Evol. 2025 Jul 21;42(7):msaf110. doi: 10.1093/molbev/msaf110 (PMC12278730; doi:10.1093/molbev/msaf110)
Supplement: msaf110_Supplementary_Data [file msaf110_supplementary_data.zip › SM_20250508.pdf]

Supplementary Material

Table S1 Sample details for 17 nine-spined stickleback populations and the ancestral population used for derived allele repolarization. Abbreviations: N_e – effective population size, F_{ROH} – the proportion of the autosomal genome in runs of homozygosity longer than 100 kb, indicating the level of inbreeding.

Population	Latitude	Longitude	Habitat	Sample size	Region	*Contemporary* N_{e*}	*Historical* N_{e*}	F_{ROH}
FIN.KAR	66.656	26.440	Pond	20	Finland	11125	11341	0.030
FIN.KEV	69.757	27.012	Lake	20	Finland	10503	1351	0.565
FIN.KRK	66.437	29.135	Pond	20	Finland	870	1263	0.624
FIN.PUL	70.017	28.018	Lake	17	Finland	1931	1712	0.496
FIN.PYO	66.261	29.433	Pond	31	Finland	144	511	0.912
FIN.RII	68.109	23.571	Lake	23	Finland	96	5139	0.442
FIN.RYT	66.384	29.320	Pond	21	Finland	761	1860	0.439
FIN.UKO	68.776	27.436	Lake	22	Finland	128559	7843	0.051
RUS.BOL	66.295	33.366	Pond	20	Russia	-	20075	0.015
RUS.KRU	66.298	33.345	Pond	20	Russia	7050	12761	0.076
RUS.LEV	66.290	33.434	Marine	30	Russia	23480	38362	0.009
RUS.MAS	66.291	33.381	Pond	21	Russia	4591	10765	0.061
SWE.ABB	64.478	19.436	Pond	21	Sweden	239	3401	0.205
SWE.BYN	64.455	19.444	Pond	23	Sweden	340	1917	0.462
SWE.HAN	64.556	19.173	Pond	20	Sweden	1188	1585	0.493
SWE.KIR	67.896	20.086	Pond	15	Sweden	824	4318	0.295
SWE.NAV	64.564	19.198	Pond	20	Sweden	730	619	0.850
JAP.BIW (ancestor)	43.082	145.113	Marine	24	Japan	13125	34099	0.117

*Obtained from Feng et al. (2023), historical N_e reflected the estimated effective population size at 1,000 years before present.

Table S2 Categorization of putative deleterious mutations from SnpEff annotations

SnpEff annotation	Deleteriousness	Impact level
splice_acceptor_variant&intron_variant	Loss-of-function (LoF)	Strongly deleterious
splice_donor_variant&intron_variant	LoF	Strongly deleterious
start_lost	LoF	Strongly deleterious
stop_gained	LoF	Strongly deleterious
stop_gained&splice_region_variant	LoF	Strongly deleterious
stop_lost	LoF	Strongly deleterious
stop_lost&splice_region_variant	LoF	Strongly deleterious
missense_variant	Missense	Mildly deleterious
missense_variant&splice_region_variant	Missense	Mildly deleterious
splice_region_variant&synonymous_variant	Synonymous	Slightly deleterious
synonymous_variant	Synonymous	Slightly deleterious

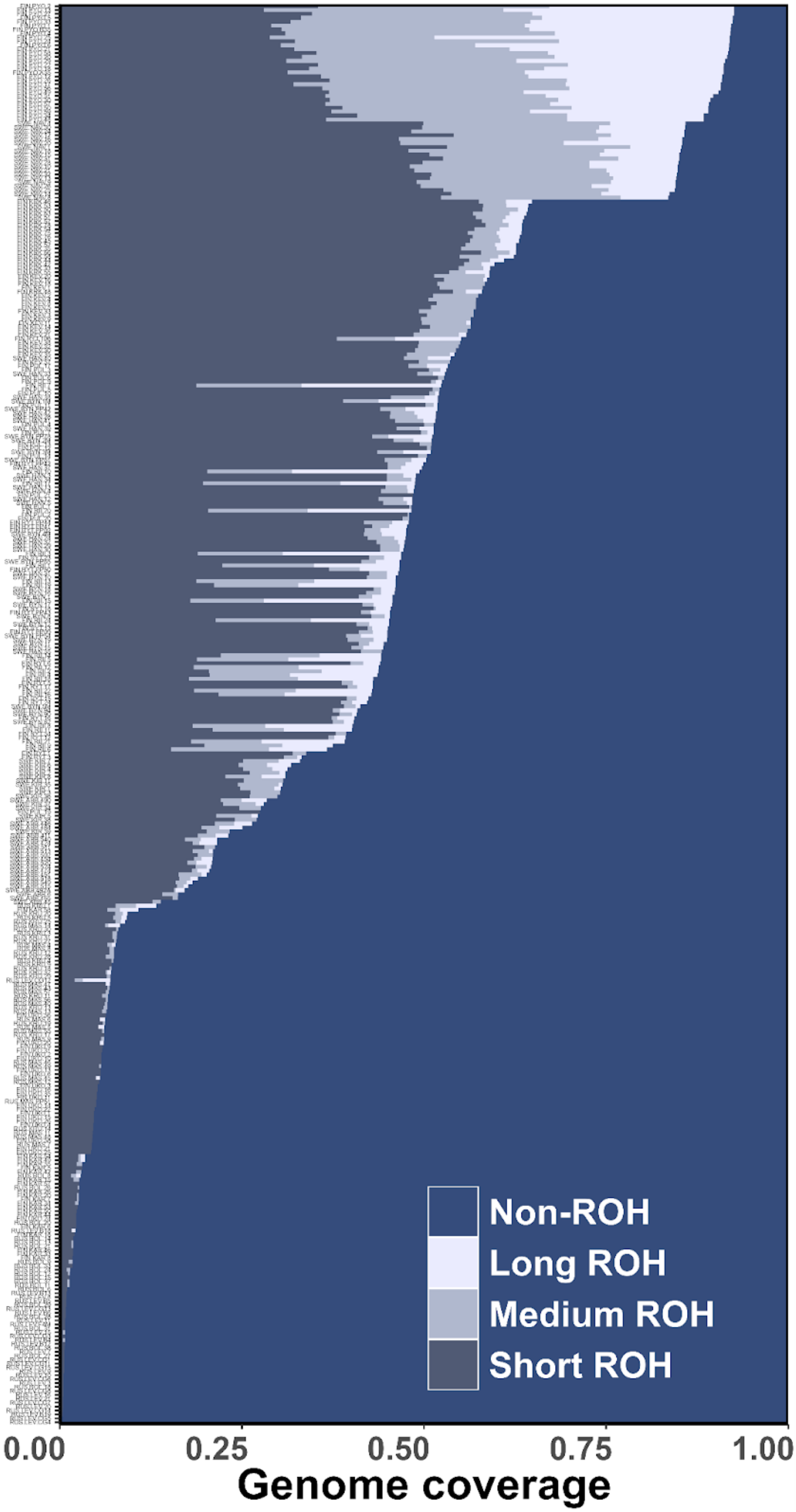

Non-ROH
Long ROH
Medium ROH
Short ROH
0.00
0.25
0.50
0.75
1.00
Genome coverage

Figure S1. The individual genome coverage of runs of homozygosity (ROH) region was divided into short (100 kb < length *(L)* < 1 Mb), medium (1 Mb < $L \leq 2$ Mb), and long (2 Mb < L) categories, as well as non-ROH regions. The individuals (n = 363) are arranged on the basis of their F_{ROH} values in ascending order from smallest to largest.

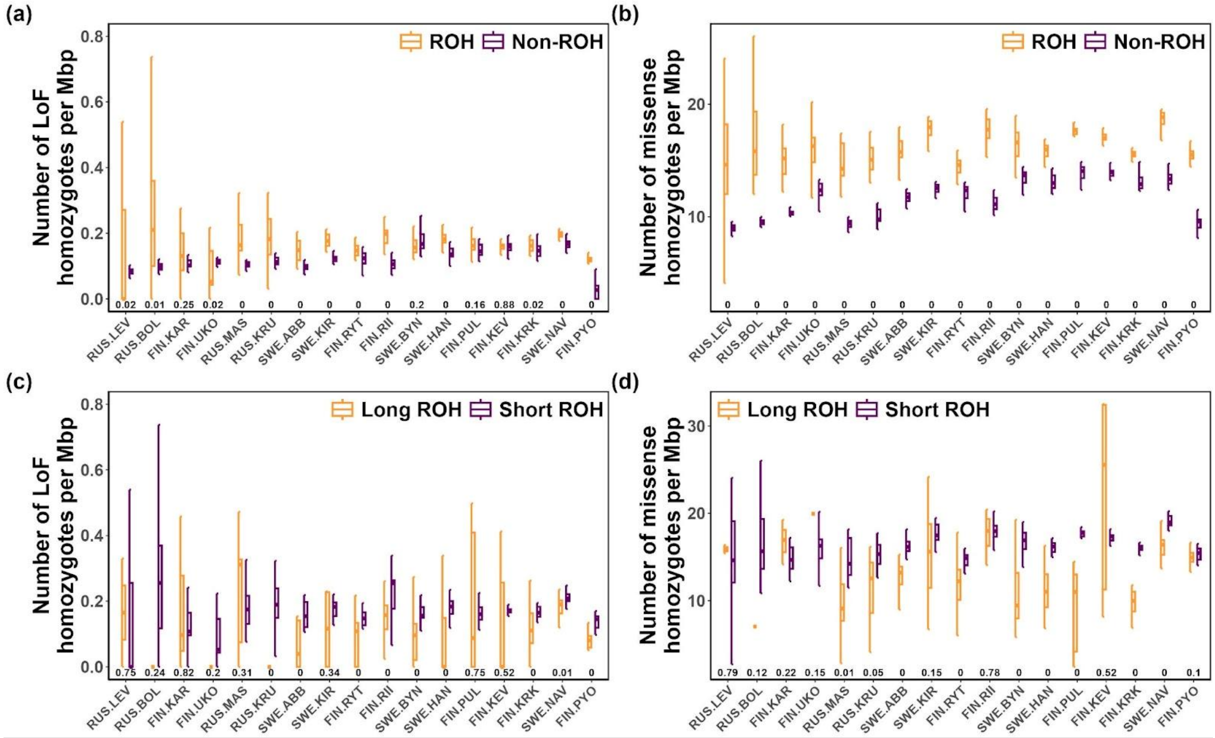

Figure S2. The number of LoF or missense homozygotes falling (a, b) in or outside ROH regions and (c, d) in long or short ROH regions per population. These estimates were normalized by the length of ROH or non-ROH and long or short ROH regions, respectively, to eliminate bias introduced by length variation. The populations were arranged in order of their inbreeding level F_{ROH} from small to large. A *p* value of less than 0.05 at the bottom of the panels indicates that there were significant differences in the mean number of LoF/missense homozygotes either between ROH and non-ROH regions or between long and short ROH regions. The *p* values of subplots a, c and d were obtained from the Mann–Whitney test, as the related response variables were not normally distributed. In the case of subplot b, where the response variable fit a normal distribution, the *p* values were obtained from Welch's two-sample *t* test.

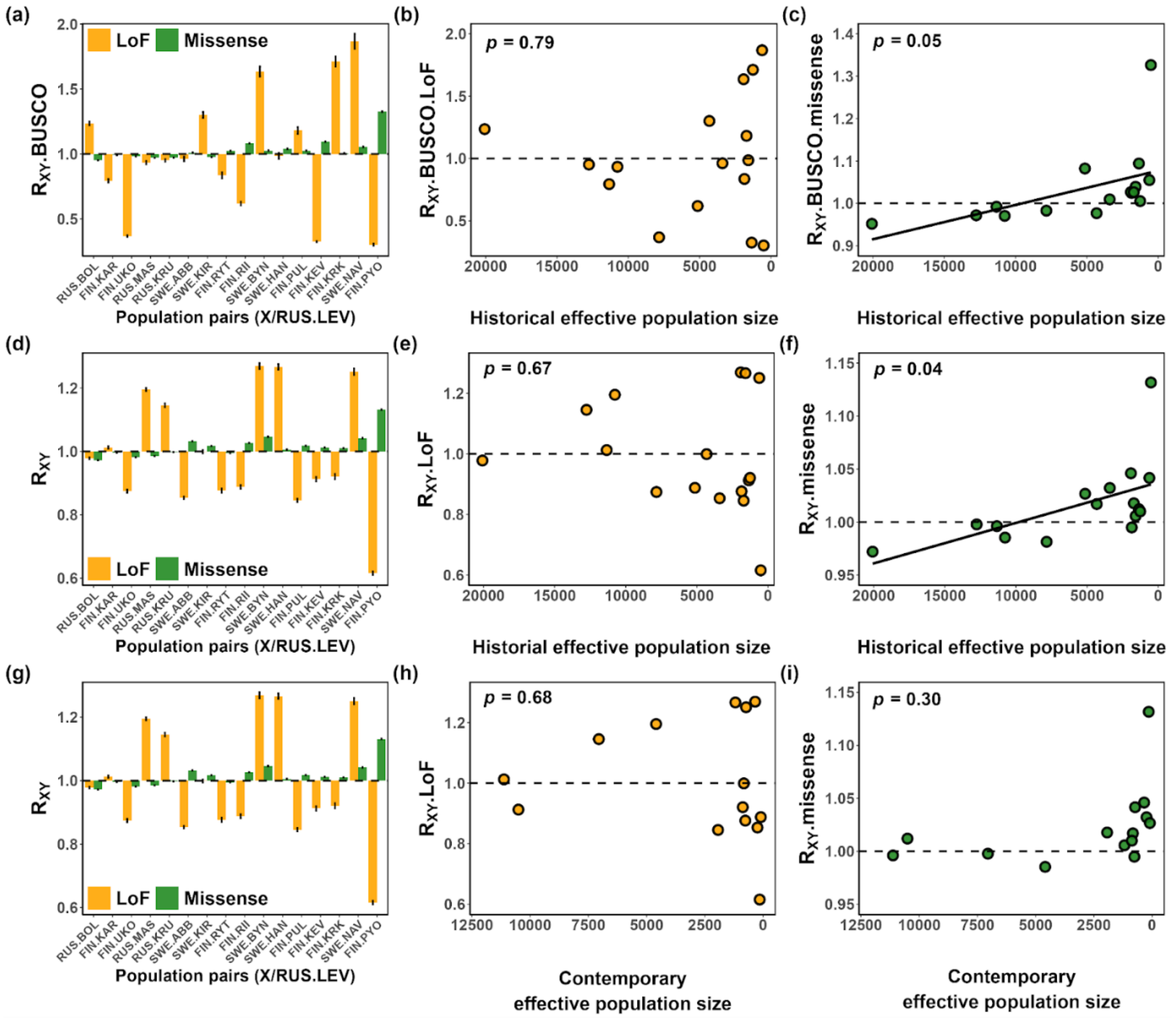

Figure S3. The relative mutation load R_{XY} matrix, calculated from variants located within benchmarking universal single-copy ortholog (BUSCO) genes (a) and relationship between the $R_{XY.\mathrm{BUSCO}}$ matrix and historical effective population sizes (estimates from Feng et al. 2023) for (b) LoF variants and (c) missense variants; (d/g) the relative mutation load R_{XY} matrix, calculated from all protein-coding variants, and relationship between R_{XY} matrix and historical effective population sizes for (e) LoF variants and (f) missense variants; between R_{XY} matrix and contemporary effective population sizes for (h) LoF variants and (i) missense variants. The error bars of R_{XY} or $R_{XY.\mathrm{BUSCO}}$ represent 95% confidence intervals estimated by the jackknifing values across the 20 autosomal linkage groups, and R_{XY} or $R_{XY.\mathrm{BUSCO}}$ indicates deficiency (<1) or excess (>1) of deleterious alleles in a given isolated freshwater population relative to that in an outbred marine population. The p values in subplots (b-c), (e-f) and (h-i) were derived from generalized linear mixed-effect models that take into account phylogenetic non-independence among populations. The populations in subplots b-c, e-f & h-i, listed from left to right, were RUS.BOL, FIN.KAR, RUS.MAS, RUS.KRU, FIN.UKO, SWE.ABB, SWE.KIR, FIN.RII, FIN.RYT, SWE.BYN, FIN.PUL, SWE.HAN, FIN.KEV, FIN.KRK, SWE.NAV, FIN.PYO.

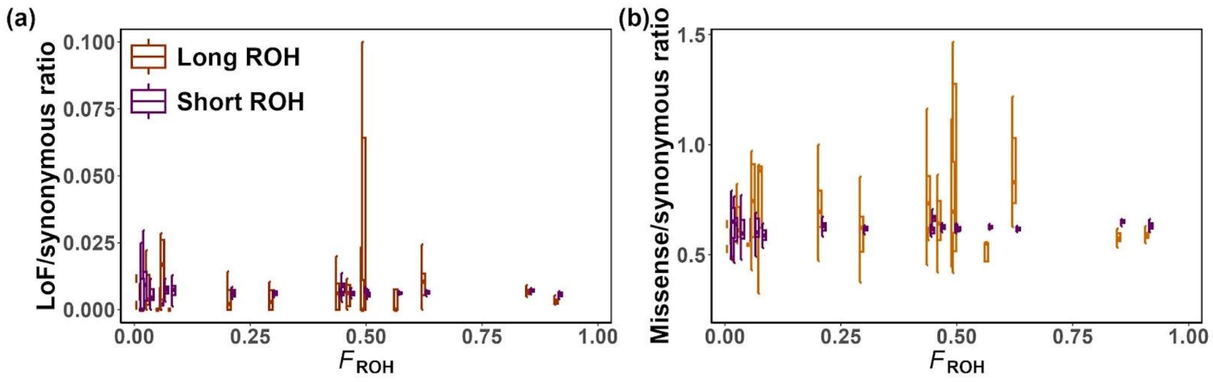

Figure S4. The efficacy of purifying selection estimates at the population level, as indicated by the ratio of deleterious to synonymous homozygous variants, for purging (a) loss-of-function (LoF) homozygotes and (b) missense homozygotes in long or short ROH.

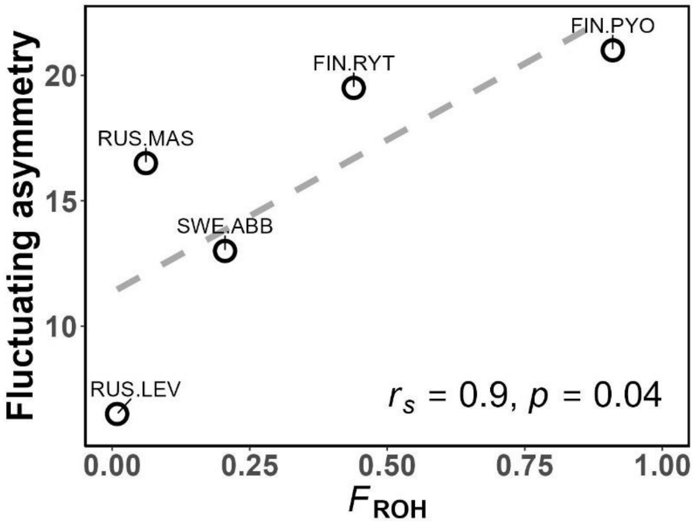

Figure S5. Relationship between fluctuating asymmetry and the level of inbreeding F_{ROH} across five populations. The *p* value was obtained from a one-sided Spearman rank correlation test since we had a priori reason to expect a positive association. Fluctuating asymmetry values were obtained from Trokovic et al. (2012).

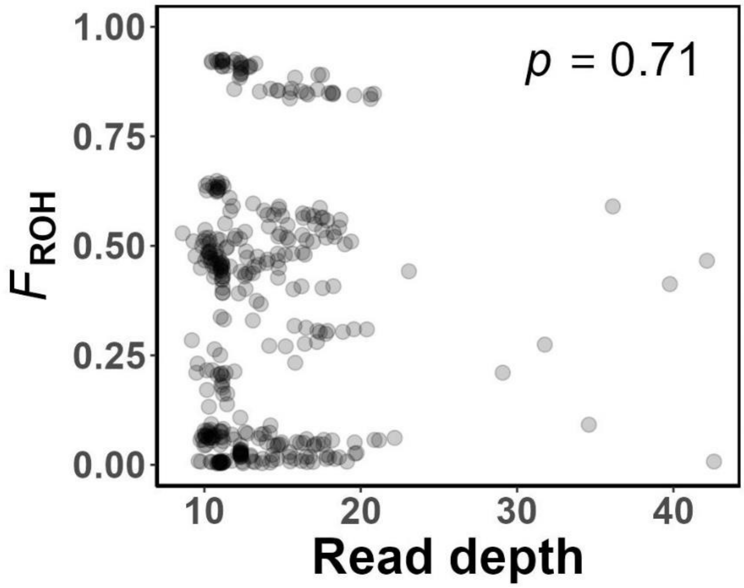

Figure S6. The individual inbreeding level, estimated as the proportion of the autosomal genome in runs of homozygosity (F_{ROH}), plotted against the read depth. The figure shows that sequencing depth does not influence F_{ROH} estimates, causing them to be biased (cf. Kardos and Waples 2024).

References

Feng X, Loytynoja A, Merila J. 2023. Estimating recent and historical effective population size of marine and freshwater sticklebacks. bioRxiv:2023.2005. 2022.541730.

Kardos M, Waples RS. 2024. Low-coverage sequencing and Wahlund effect severely bias estimates of inbreeding, heterozygosity and effective population size in North American wolves. Molecular Ecology:e17415.

Trokovic N, Herczeg G, Ghani NIA, Shikano T, Merilä J. 2012. High levels of fluctuating asymmetry in isolated stickleback populations. BMC Evolutionary Biology 12:1-9.
