# Supplementary figures and images for: Dynamics of Deleterious Mutations and Purifying Selection in Small Population Isolates
Source: Mol Biol Evol. 2025 Jul 21;42(7):msaf110. doi: 10.1093/molbev/msaf110 (PMC12278730; doi:10.1093/molbev/msaf110)

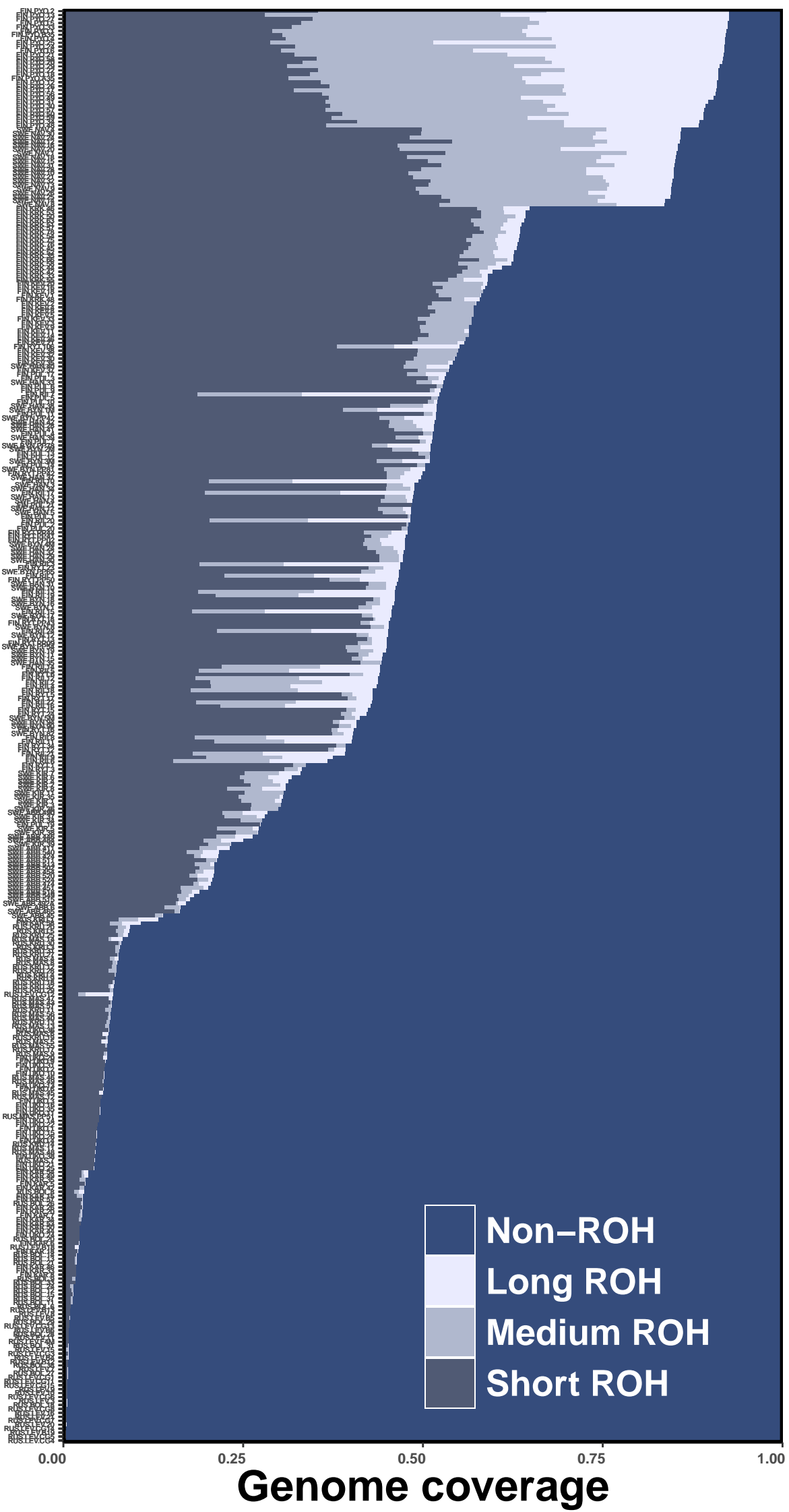

Supplement: msaf110_Supplementary_Data [file msaf110_supplementary_data.zip › Fig.S1.pdf]

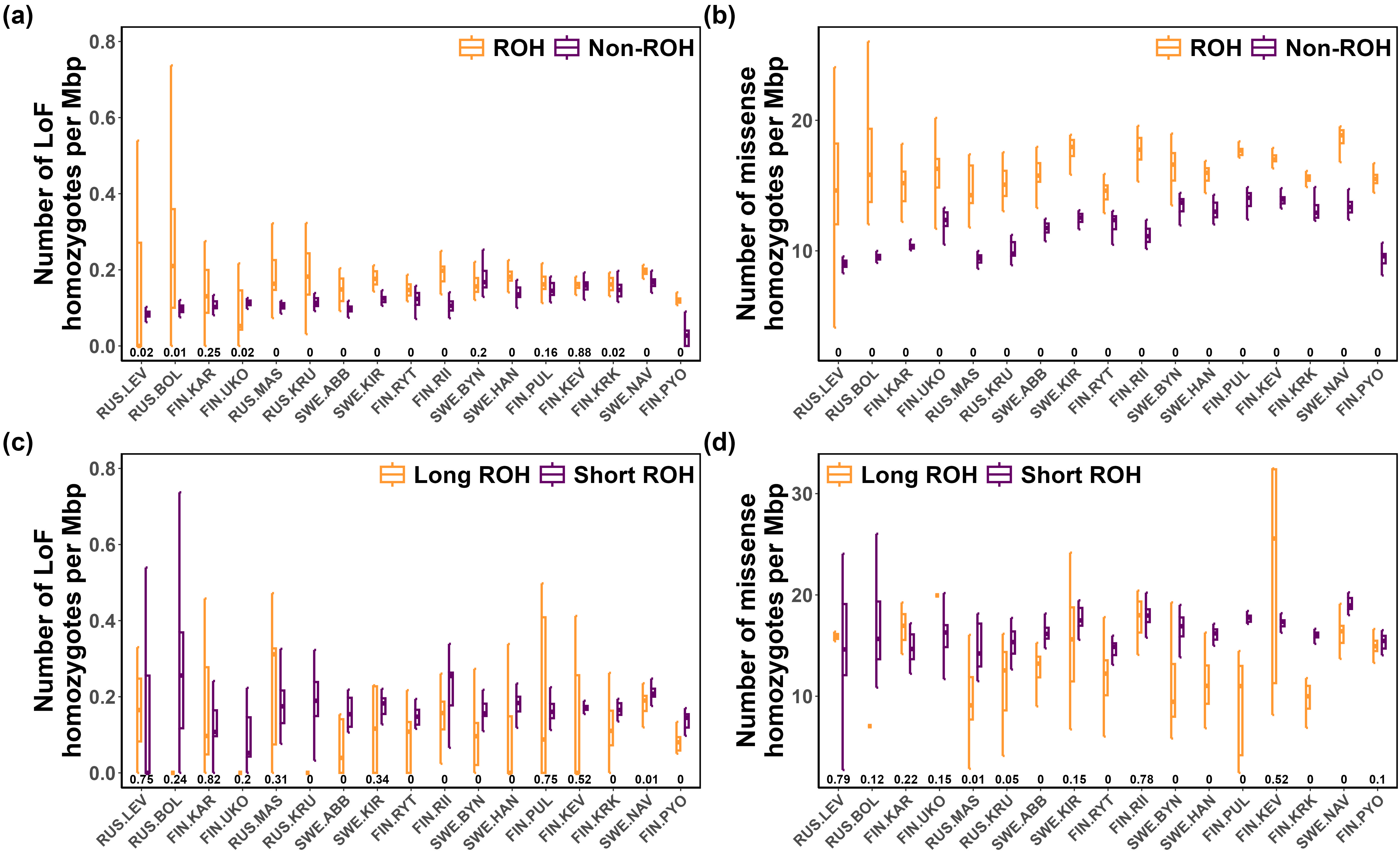

Supplement: msaf110_Supplementary_Data [file msaf110_supplementary_data.zip › Fig.S2.jpg]

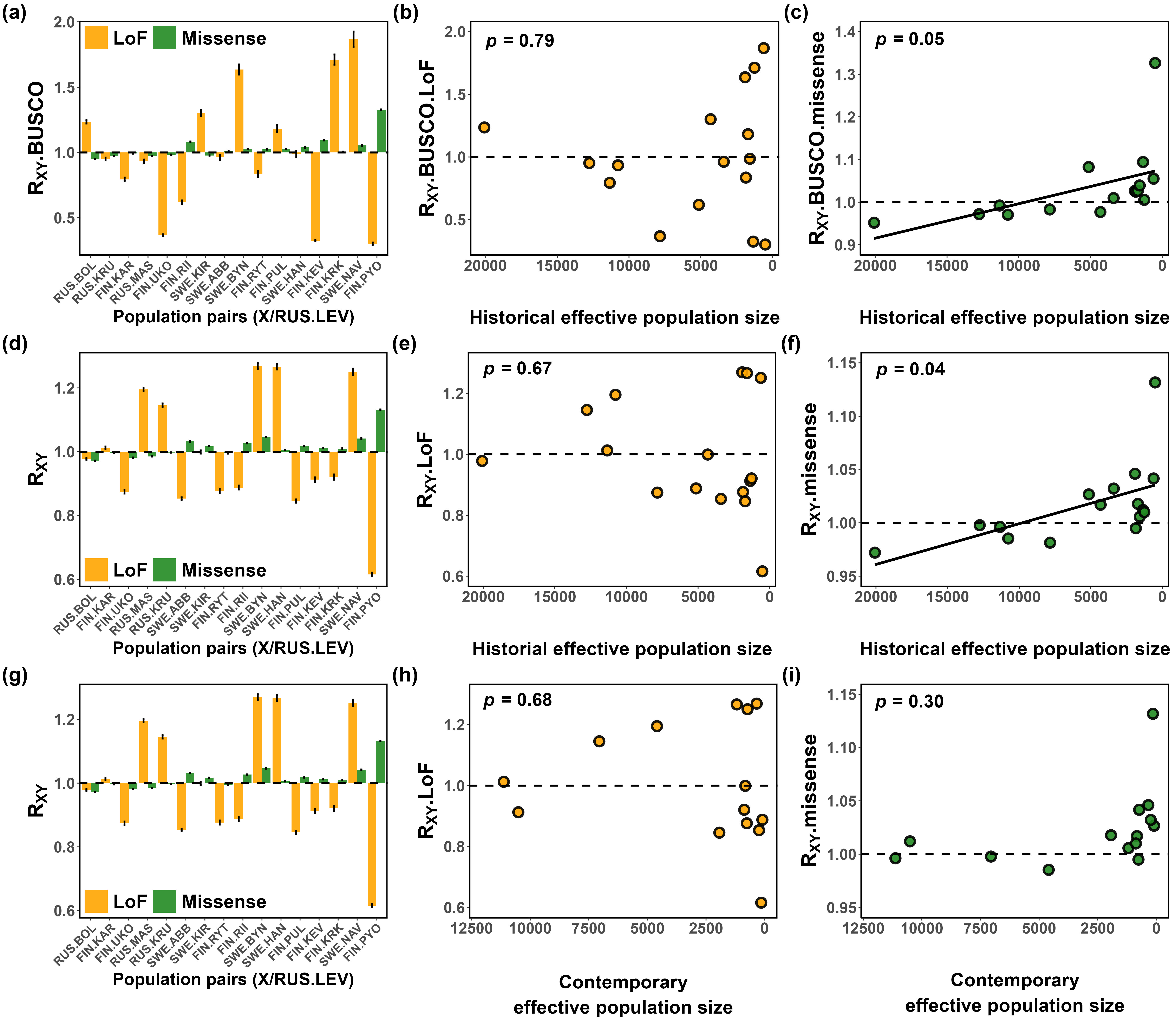

Supplement: msaf110_Supplementary_Data [file msaf110_supplementary_data.zip › Fig.S3.jpg]

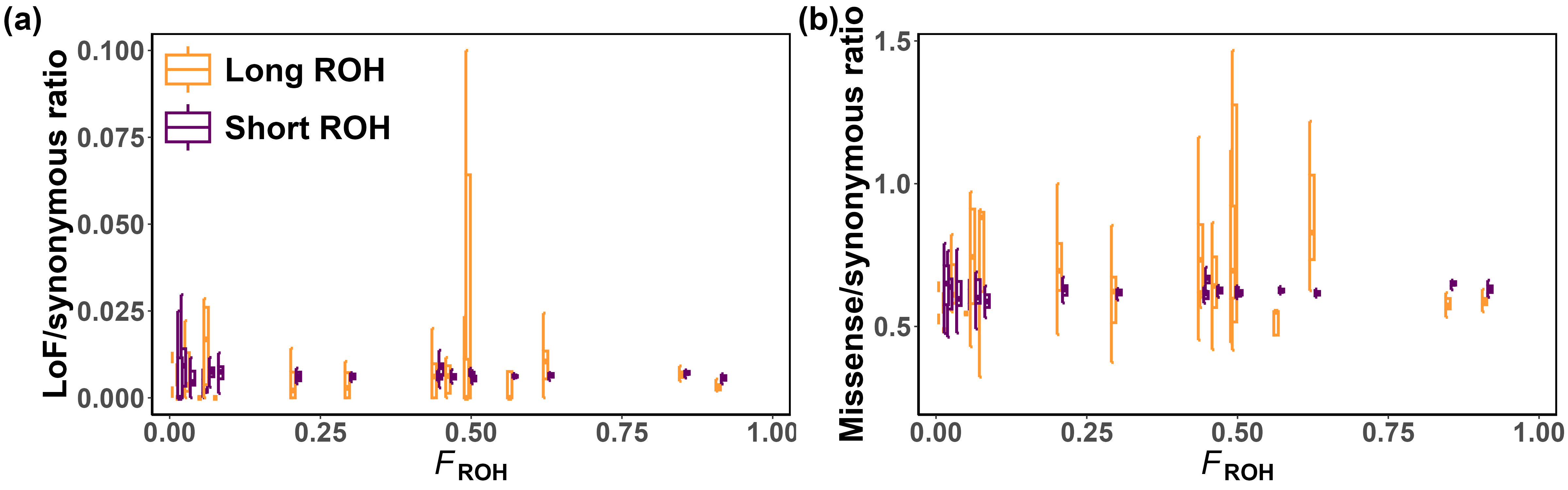

Supplement: msaf110_Supplementary_Data [file msaf110_supplementary_data.zip › Fig.S4.jpg]

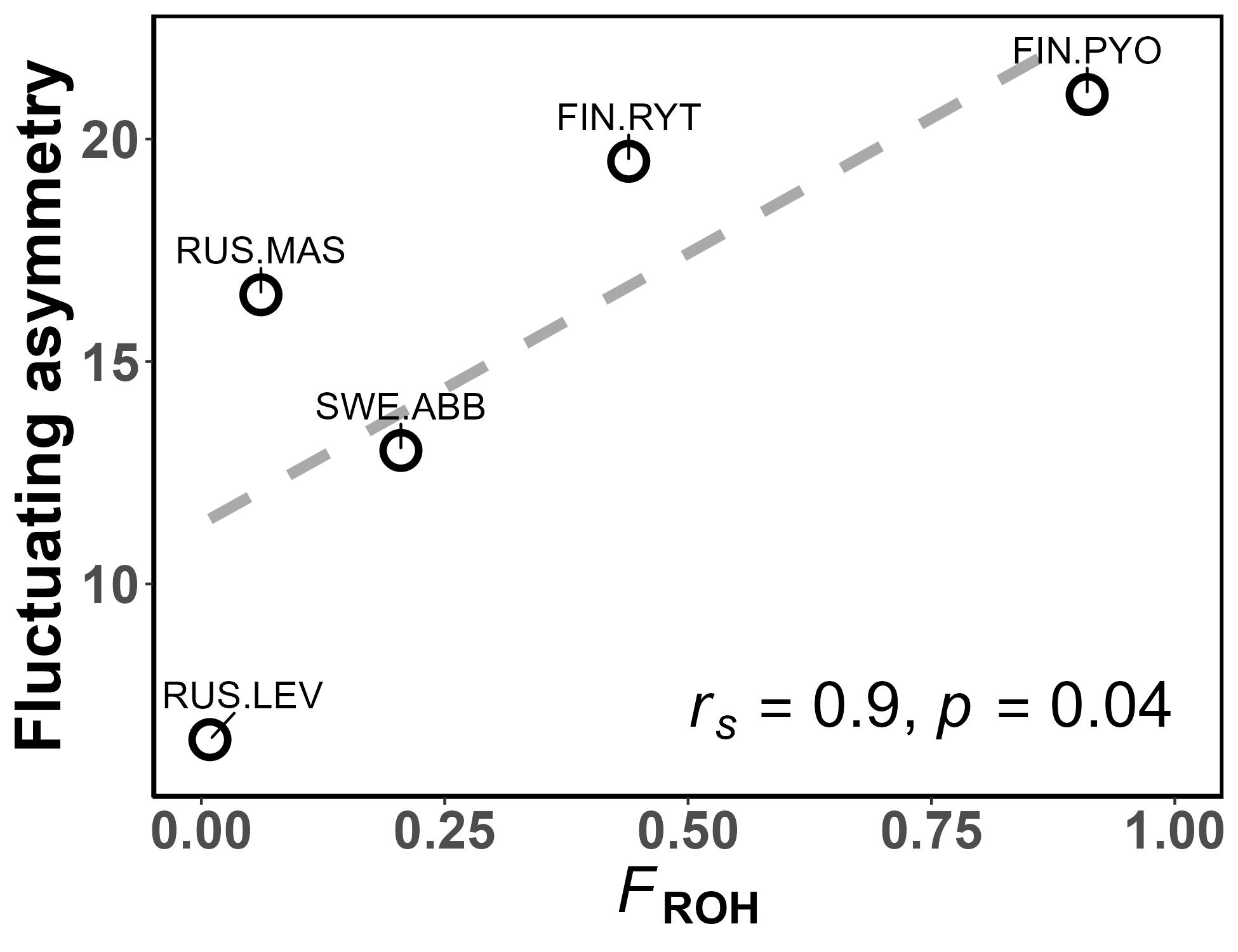

Supplement: msaf110_Supplementary_Data [file msaf110_supplementary_data.zip › Fig.S5.jpg]

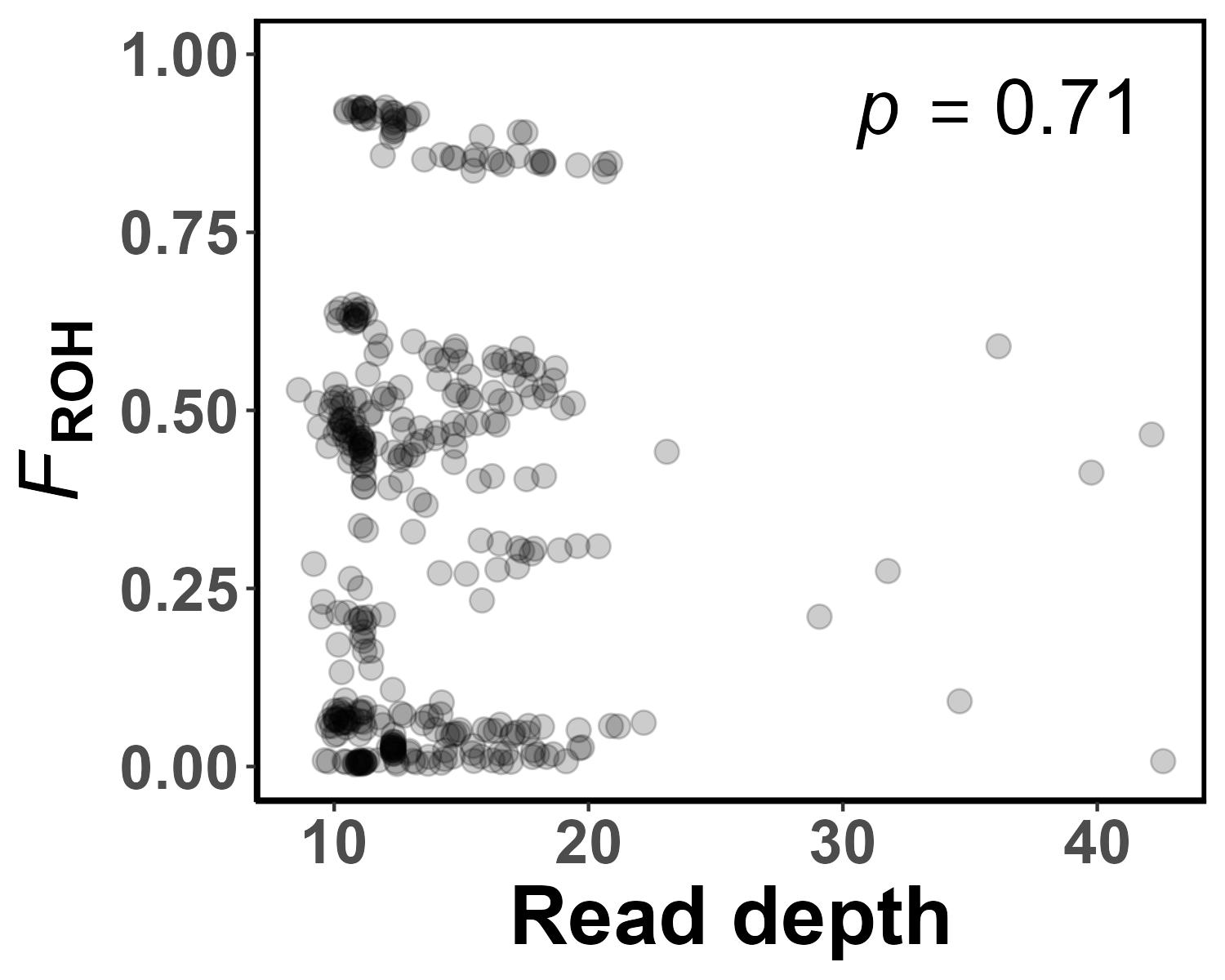

Supplement: msaf110_Supplementary_Data [file msaf110_supplementary_data.zip › Fig.S6.jpg]
